# Series of Protonated Nitrogen Bases with a Weakly Coordinating Counteranion: Observation of the ^14^N–^1^H Spin–Spin Coupling

**DOI:** 10.1021/acsorginorgau.3c00045

**Published:** 2023-10-20

**Authors:** Maria C. Carrasco, Firoz Shah Tuglak Khan, Shabnam Hematian

**Affiliations:** Department of Chemistry and Biochemistry, University of North Carolina at Greensboro, Greensboro, North Carolina 27402, United States

**Keywords:** Protonated nitrogen bases, NMR spectroscopy, Nitrogen-14 nuclear quadrupole effects, Solvent effects, Ion pairing

## Abstract

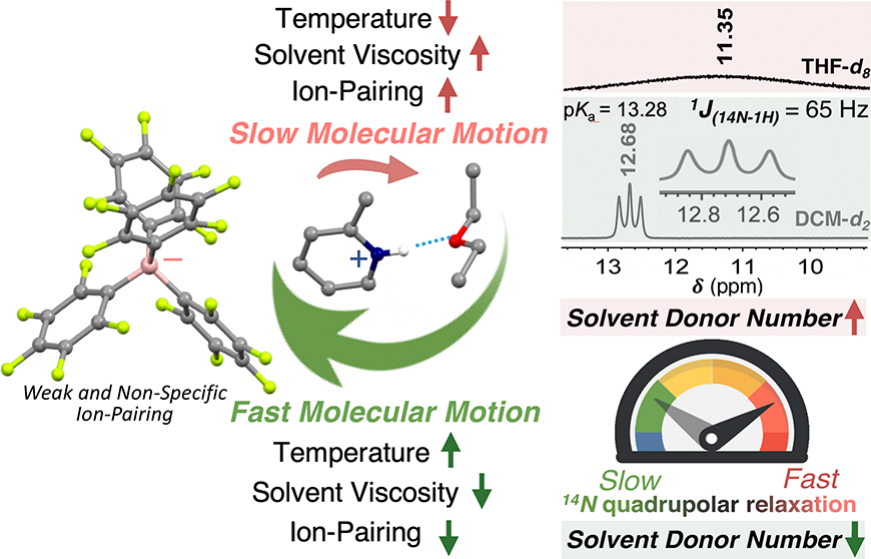

A distinguished triplet splitting pattern for the ^14^N–^1^H couplings in the proton signals of a series of protonated nitrogen bases—aliphatic and aromatic amines, as well as pyridines—with the weakly coordinating tetrakis(pentafluorophenyl)borate anion, [B(C_6_F_5_)_4_]^−^, is observed for the first time in nonaqueous media at room temperature. The effects of ion pairing, solvent parameters, and correlation between the δ_H_, ^1^*J*_NH_, and p*K*_a_ values are reported.

Nitrogen-containing molecules are ubiquitous in nature and play vital roles in many biological and chemical processes.^1,2^ Many nitrogen functional groups can serve as a base accepting a proton and, thus, are often involved in proton transfer reactions.^3^

The proton donor–acceptor affinities of these molecules are among the most tunable and can be significantly altered by modifying their molecular and electronic structures.^3−7^ Thus, many protonated nitrogen species are used as the weak proton source in proton transfer reactions in organic solvents and ionic liquids.^8,9^ They are widely used in proton-coupled electron transfer (PCET) reactions where the electron and proton transfer processes are intertwined in a concerted or stepwise fashion, such as those related to synthesis and catalysis.^10−15^ Alternatively, these weak protonated nitrogen acids provide a measure for determining p*K*_a_ values of basic sites in a variety of systems, particularly many reactive intermediates (e.g., oxo or peroxo species) in low-temperature studies that are generally conducted in lower-polarity solvents.^16−21^ However, depending on the counteranion used and the ion pairing in solution, the utilization of such protonated nitrogen acids can be limited because of undesirable nucleophilicity, solubility, or chemical stability.^22−24^

Apart from their roles in acid–base reactions, nitrogen-14 sites in molecules are of broad interest for the study of intermolecular interactions because of their widespread occurrence in chemical and biological systems. The quadrupolar ^14^N coupling constants are particularly sensitive to changes in the geometry at the nitrogen site and may be used as a tool to investigate molecular properties and structures.

In this study, the bulky and weakly coordinating counterion tetrakis(pentafluorophenyl)borate, [B(C_6_F_5_)_4_]^−^, is employed for the preparation of a series of protonated nitrogen bases—aliphatic and aromatic amines, as well as pyridines—through either salt metathesis or acid–base reactions (Scheme S1).^25^ All of our synthetic and characterization procedures were performed in a broad range of organic solvents under rigorous dry and air-free conditions.

The solution structures of the [B(C_6_F_5_)_4_]^−^ salts of all eight protonated nitrogen bases were studied by ^1^H and ^19^F NMR spectroscopies in deuterated dichloromethane (DCM) (Figures S1–S17). There is a visible trend between N–H proton signal shifts and the known p*K*_a_ values of such protonated nitrogen species.^4,26−28^ The systems discussed in this study span a p*K*_a_ range from 11.5 to 18.8 in MeCN. Within each class of the N–H bonds with similar hybridization (i.e., sp^3^ in ammoniums, δ = 5.44–6.50 ppm, and anilinium, δ = 8.81 ppm, along with sp^2^ in pyridiniums, δ = 12.13–12.68 ppm), the more acidic protons resonate at a lower field.

Strikingly, the ^1^H NMR spectra of all of these protonated nitrogen systems also exhibit a distinguished triplet splitting pattern for the coupling of the acidic proton to the quadrupolar ^14^N nuclei (99.7% abundant, *I* = 1; see Figure 1a,b). This is surprising because the signals for this class of protons are usually either not observable or significantly broadened because of rapid chemical exchange and/or rapid ^14^N quadrupole relaxation.^29−31^

**Figure 1:**
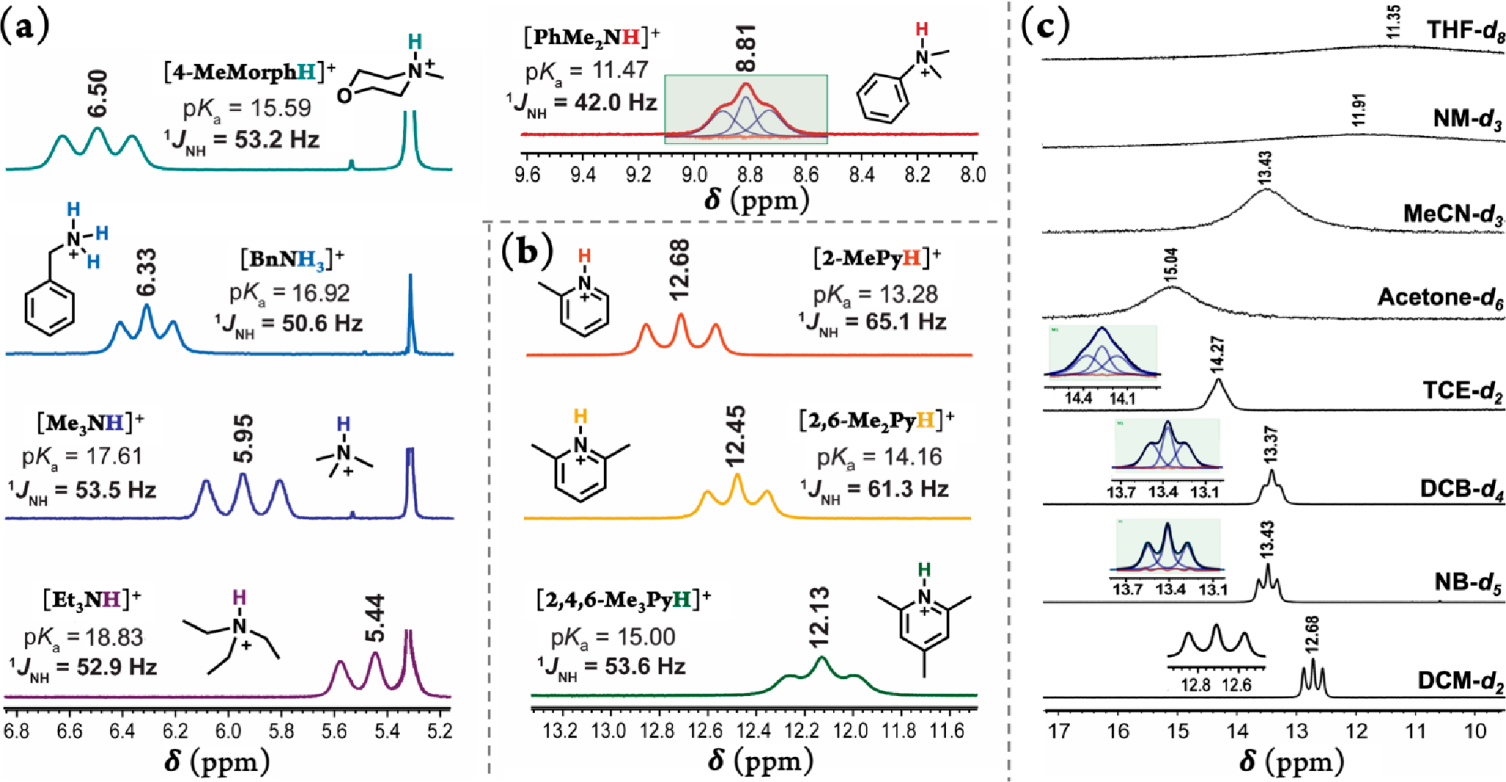
Part of the ^1^H NMR spectra of the [B(C_6_F_5_)_4_]^−^ salts of (a) the protonated aliphatic and aromatic amines, as well as (b) protonated pyridines displaying the ^14^N–^1^H spin–spin coupling in DCM-*d*_2_ at room temperature. The ^1^H NMR spectra of (c) [2-MePyH][B(C_6_F_5_)_4_] in a series of deuterated solvents at room temperature. The disappearance of N–H splitting in the solvents with greater donor numbers reflects the increase in ^14^N relaxation rates. The line shape fittings are shown in green boxes. The listed p*K*_a_ values in MeCN were obtained from the literature.^4,6^

Quadrupolar relaxation of the ^14^N nucleus is typically rapid in the presence of a large electric field gradient at the nitrogen site. In such systems, ^14^N–^1^H coupling is often not observed because the signal of the proton interacting with the ^14^N nucleus is broadened or decoupled by its quadrupolar relaxation. However, in a highly symmetrical molecule (e.g., ammonium ion with cubic symmetry, NH_4_^+^, ^1^*J*_NH_ = 51.5 Hz, or ammonia with 3-fold symmetry, NH_3_, ^1^*J*_NH_ = 43.8 Hz), with only very small fluctuation in the electric field at the ^l4^N nucleus, the relatively slower quadrupolar spin–lattice relaxation leads to a 1:1:1 triplet line proton signal because the proton can “see” the three nitrogen magnetic quantum states, and ^14^N–^1^H coupling becomes observable.^30,31^

For systems with lower symmetry there are currently limited examples of direct measurement of such ^14^N–^1^H coupling over one bond using conventional ^1^H NMR spectroscopy.^32−39^ Additionally, advanced NMR techniques, such as rotating frame, frequency-selective pulse, or solid-state measurements under fast magic-angle spinning, have been used to observe the ^14^N–^1^H coupling over one bond.^40−43^ Other reports of triplet splitting in solution ^1^H NMR studies were described for long-range couplings of ^1^H and ^14^N nuclei through two (i.e., geminal) or three (i.e., vicinal) bonds in a variety of quaternary ammonium, pyridinium, and pyrazinium salts.^38,44−48^

However, in all these reports the ^14^N–^1^H splittings only appeared in aqueous solutions, and the use of strongly acidified solution along with elevated temperatures to increase the molecular motions were necessary to observe the coupling.^32−39,44−48^ Expectedly, the disappearance of the triplet splitting in nonaqueous solutions has long been ascribed to the effect of ion pairing both on the field gradient at nitrogen and on the molecular correlation times.^30^

Here, for the first time, we observe the ^14^N–^1^H couplings over one bond in nonaqueous solutions at room temperature. The coupling constants observed for all four protonated aliphatic amines (i.e., [Et_3_NH]^+^, [Me_3_NH]^+^, [4-BnNH_3_]^+^, and [4-MeMorphH]^+^) are in the range of 50.6–53.5 Hz, which is characteristic for the coupling magnitude of ^14^N–^1^H for directly bonded hydrogen to a nitrogen with sp^3^ hybridization (Table S1).

Of note in the spectrum of 4-methylmorpholinium are also the four unique proton signals corresponding to two sets of axial and two sets of equatorial protons in the ring; by comparing the NMR spectra of the acid and base forms (Figures S5 and S18), it is evident that the protonation blocks or slows down the chair flip in these molecules.

Additionally, the dimethylanilinium with the lowest p*K*_a_ value shows a broad triplet signal with a coupling constant of 42.0 Hz^49^ which was obtained from line shape fitting by constraining the three lines of equal areas and 1.5 times greater broadening (i.e., line width) for the outer lines as for the central one (Figure 1a).^50^

The triplets for the pyridinium class also demonstrate a 1:1:1 splitting pattern and appear symmetrical in peak intensity, with [2,4,6-Me_3_PyH]^+^ exhibiting the more noticeable peak broadening. As expected, because of more s character at the ^14^N atom (i.e., sp^2^), larger ^1^*J*_NH_ values were observed in pyridinium species. The magnitudes of coupling found for these were 53.6, 61.3, and 65.1 Hz for [2,4,6-MePyH]^+^, [2,6-Me_2_PyH]^+^, and [2-Me_3_PyH]^+^, respectively. The observed progression is in agreement with the 69 Hz value reported for the parent pyridinium.^33−35^ Here, a noticeable trend between the ^14^N–^1^H coupling constants and p*K*_a_ values was also observed; the increased acidity of the exchangeable protons correlates with greater spin–spin coupling interactions. This relationship, along with the proton chemical shifts, can serve as a probe of molecular basicity/acidity, which is particularly important for nonaqueous media.

We hypothesized that the observation of the ^14^N–H splittings for these systems in nonaqueous media partially stems from a significantly weaker or nonspecific ion pairing interaction^51^ provided by [B(C_6_F_5_)_4_]^−^, which can decrease the field gradient at nitrogen and increase the molecular rotation in the solution. This proposition was further supported by a comparison of the ^14^N–H coupling interactions in [Et_3_NH]^+^ when paired with either [B(C_6_F_5_)_4_]^−^ or the more coordinating [SbF_6_]^−^ counterion. The latter exhibited a more severe quadrupolar broadening of the proton signal because of the specific and stronger ion pairing interaction (Figure S19–S22).^25^

To further understand the role of the interactions between the solvent and solute, we also carried out ^1^H NMR measurements for [2-MePyH][B(C_6_F_5_)_4_]·Et_2_O in a series of deuterated solvents (Figures 1c and S23–S29). The relevant parameters for the solvents chosen for this study are given in Table 1.

**Table 1:** Relevant Solvent Parameters^a^

solvents	ε	μ(D)	DN	AN	*η*(mPa·s)
nitromethane (NM)	36.7^b^	3.57^b^	2.7^b^	20.5^b^	0.65^e^
MeCN	36.0^b^	3.44^b^	14.1^b^	18.9^b^	0.38^e^
nitrobenzene (NB)	34.8^b^	4.0^b^	4.4^b^	14.8^b^	2.03^f^
acetone	20.7^b^	2.88^b^	17.0^b^	12.5^b^	0.32^e^
DCB	9.9^c^	2.50^d^	3.0^e^	8.5^c^	1.43^f^
DCM	8.9^b^	1.5^b^	∼0^b^	20.4^b^	0.43^e^
TCE	8.2^c^	1.31^d^	∼0^c^	10.3^c^	1.80^f^
THF	7.4^b^	1.7^b^	20^b^	8.0^b^	0.55^e^

a Dielectric constant (ε); dipole moment (μ); donor number (DN); acceptor number (AN); absolute viscosity (η).

b Reference (52).

c Reference (53).

d Reference (54).

e Reference (55).

f Reference (56).

Among all of the nonaromatic solvents studied here, the ^14^N–H coupling interactions were only observed in deuterated DCM and, unsurprisingly, to a smaller extent in 1,1,2,2-tetrachloroethane (TCE) with the relatively higher viscosity (Figure 1c). Generally, in low-viscosity solvents, the molecular motions are faster, thus, longer ^14^N quadrupolar relaxation times and better resolved triplets are expected.^30^

The results indicate that other factors, such as solvent donor and acceptor numbers, can play a more significant role in modulating ^14^N quadrupole relaxation. Here, the positively charged protonated nitrogen species experience greater intermolecular associations and solvation in solvents with higher donor numbers (e.g., acetone or THF). In turn, that leads to slower molecular reorientation, faster ^14^N quadrupolar relaxation, and the disappearance of the ^14^N–H splitting. This is in excellent agreement with our recent report on faster molecular motions for other monocationic systems, such as ferriceniums, with weakly coordinating anions in DCM.^57^

Interestingly, the ^14^N–H coupling is also to some extent observable in the two aromatic solvents studied here, i.e., nitrobenzene (NB) and 1,2-dichlorobenzene (DCB) (Figure 1c). This is consistent with the reported faster and less hindered rotation of the molecules in aromatic solvents.^58^ Between these two aromatic solvents, NB, with a higher dielectric constant, leads to less significant ion pairing and longer ^14^N quadrupolar relaxation times.

Next, to further study the molecular structures of the protonated nitrogen bases in solid state, crystals suitable for X-ray crystallography of six [B(C_6_F_5_)_4_]^−^ salts were obtained from the concentrated diethyl ether solutions at −30 °C.^25^ Full crystallographic details are provided within Tables S2 and S3. The protons attached to the nitrogen atoms were located in difference Fourier maps and were refined isotopically.^25^ In all six structures, a moderate hydrogen-bonding interaction^59^ between the protonated nitrogen and oxygen atom of a diethyl ether molecule (Et_2_O···H–^+^N) was present (i.e., N···O: 2.67–2.80 Å; Figure 2).

**Figure 2:**
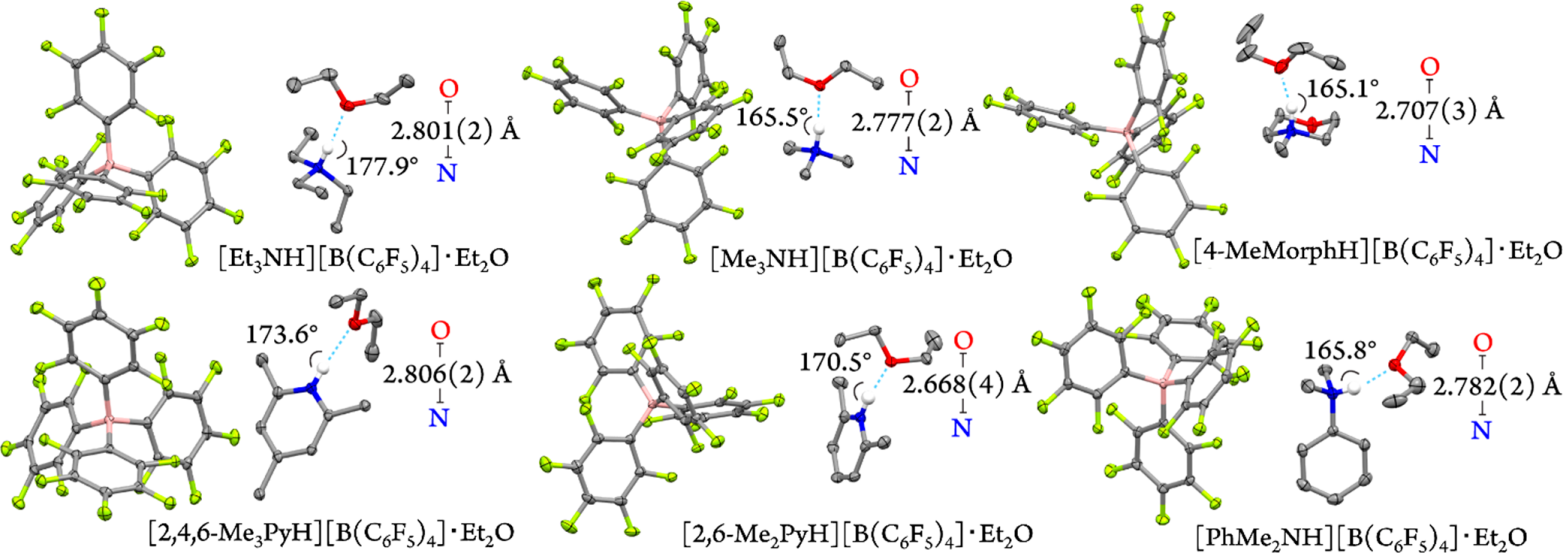
Displacement ellipsoid plot (50% probability level) of the six protonated nitrogen species at 100(2) K. All of the hydrogen atoms, except for the acidic proton, have been omitted for clarity. Dotted lines show the H-bonding interactions between the protonated nitrogen species and diethyl ether molecules.

Interestingly, within the aliphatic systems, the longest N···O distance was observed for the weakest acid (i.e., ∼2.80 Å in triethylammonium). A similar trend was observed within the pyridiniums, as the stronger acid, 2,6-dimethylpyridinium, maintains the shorter N···O distance relative to 2,4,6-trimethylpyridinium.

The average C–N bond distances increase upon protonation (Table S4). The elongation of the C–N bonds is most pronounced in the aromatic amine, PhMe_2_N (ΔC–N ≈ 0.07 Å) followed by the aliphatic systems, Et_3_N, Me_3_N, and 4-MeMorph (ΔC–N ≈ 0.03 Å), while the protonation leads to little to no change in the C–N bond distances in pyridine analogues. The C–N–C bond angle has also been found to slightly increase (i.e., by ∼1°) upon protonation in the aliphatic systems, while this widening is more significant (i.e., by ∼5°) in the pyridine derivatives. This is consistent with the trend reported for the parent pyridine/pyridinium couple (i.e., 116.6° to 122.6°).^60,61^

As expected, the protonation of the nitrogen center of *N,N*-dimethylaniline disrupts the conjugation of the nitrogen lone pair with the aryl substituent and accompanies a significant shift in hybridization (sp^2^ → sp^3^) at the nitrogen site and structural change, thus leading to a considerable decrease in the C–N–C angle (approximately −8°).

Aside from the counterion peaks, the IR spectra of all protonated nitrogen bases display sharp N–H stretching bands in the range of 3241–3367 cm^–1^ (Table S5 and Figures S37–S44). Because of the protonation, the C–H stretching frequencies for the aliphatic and aromatic systems are generally shifted to higher energies, while in pyridinium species they appear at lower energies compared with their neutral bases.^25^

In summary, this report has described the synthesis and molecular structures of a series of protonated nitrogen species with a weakly coordinating [B(C_6_F_5_)_4_]^−^ counterion. The weaker ion pairing allowed for observations of ^14^N–^1^H spin–spin coupling in nonaqueous media for the first time. We also demonstrated that in addition to viscosity and aromaticity, other solvent parameters, such as donor and acceptor numbers, directly govern the molecular motions of these charged species and can effectively influence the quadrupole relaxation of the ^14^N sites. It is notable that the observed trends for the proton chemical shifts (δ_H_) and ^14^N–^1^H coupling constants (^1^*J*_NH_) of the acidic protons also correlate well with the known p*K*_a_ values of these species and, in combination, they can be used as a proxy for acidity, which is particularly challenging in nonaqueous media. Furthermore, we propose that this coupling phenomenon and the combination of the δ_H_ and ^1^*J*_NH_ values may facilitate the discovery and characterization of important nitrogen sites in a variety of systems. For instance, the proton resonance obtained from the addition of an appropriate acid (i.e., [H(OEt_2_)_2_][B(C_6_F_5_)_4_]) to an unknown sample, such as a natural product with a nitrogen site in a suitable solvent, may provide the information necessary to identify the molecular structure. Detailed experimental work is needed to confirm our proposition; such work is currently underway in our laboratory and will be reported in due course.

Furthermore, taken together, our findings will also help lead to significant scientific advancement toward understanding molecular motions, particularly of systems containing quadruple isotopes.

## Data Availability

The data underlying this study are available in the published article and its Supporting Information.

## Supporting Information Available

Experimental procedures and IR, ^1^H, and ^19^F NMR spectroscopic and crystallographic data (PDF)
