# Supplementary material for: Series of Protonated Nitrogen Bases with a Weakly Coordinating Counteranion: Observation of the 14N–1H Spin–Spin Coupling
Source: ACS Org Inorg Au. 2023 Oct 20;4(1):91–6. doi: 10.1021/acsorginorgau.3c00045 (PMC10853991; doi:10.1021/acsorginorgau.3c00045)
Supplement: Supplementary file 1 — gg3c00045_si_001.pdf [file gg3c00045_si_001.pdf]

Supporting Information

A Series of Protonated Nitrogen Bases with A Weakly Coordinating Counteranion: Observation of the ^{14}N-^{1}H Spin-Spin Coupling

Maria C. Carrasco,‡ Firoz Shah Tuglak Khan,‡ and Shabnam Hematian*

Department of Chemistry and Biochemistry, University of North Carolina at Greensboro, Greensboro, NC 27402, USA

Materials and Methods

All chemicals and solvents were of commercially available grade and used as received unless otherwise mentioned. Acetonitrile (MeCN) was purchased from Sigma-Aldrich and Diethyl ether was purchased from Fisher Scientific. The solvents were further purified by passing through a 18- or 60-cm-long column of activated alumina under argon using an Innovative Technologies or Inert PureSolv Micro solvent purification system. The solvents were further deoxygenated by bubbling them with argon for an hour prior to sending to the glovebox. These solvents were then stored in dark glass bottles inside the glovebox over 3 or 5 Å molecular sieves for more than 72 hours prior to use. The protonated amines were synthesized and handled under an inert atmosphere using either argon and standard Schlenk line techniques or a Vacuum Atmospheres OMNI-Lab inert atmosphere (<0.5 ppm of O_2 and H_2O) glovebox filled with nitrogen.

Benzylammonium chloride (99%), and 2,4,6-collidine (99%) were purchased from Alfa Aesar. Trimethylamine hydrochloride (>98%), triethylamine hydrochloride (>98%), 2-methyl pyridine (98%), 2,6-lutidine (≥99%), *N,N*-dimethylaniline (≥99.5%), 4-methylmorpholine (≥99.5%), and silver hexafluoroantimonate(V) (98%) were purchased from Sigma Aldrich. Hydrogen chloride in diethyl ether (2 M) was purchased from Acros Organics. $K[B(C_6F_5)_4]$ and $Li[B(C_6F_5)_4](2.35\ Et_2O)$ (99.9%) were purchased from Boulder Scientific Company. Dichloromethane-d_2 (D, 99.8%), tetrahydrofuran-d_8 (D, 99.5%), acetone-d_6 (D, 99.9%), acetonitrile (D, 99.8%) were purchased in 1 g or 0.75 mL ampules from Cambridge Isotope Laboratories. Nitromethane-d_3 (D, 99%), 1,1,2,2-tetrachloroethane-d_2 (D, ≥99.5%), 1,2-dichlorobenzne-d_4 (D, 98%), and nitrobenzene- d_5 (D, 99.5%) were purchased from Sigma-Aldrich.

^{1}H-NMR and ^{19}F-NMR spectra were recorded either on a JEOL 400 or 500 MHz instrument. The residual ^{1}H resonances of the solvents were used as a reference. Infrared (IR) spectra were obtained using a Thermo Scientific Nicolet iS5 Fourier Transform IR (FT-IR) spectrometer equipped with an iD7 attenuated total reflection (ATR) accessory. Single-crystal X-ray data were collected using a Gemini R (Agilent Technologies) diffractometer at the X-ray diffraction facility of the Joint School of Nanoscience and Nanoengineering (JSNN). The temperature of the data collection was controlled using the system Cryojet (manufactured by Oxford Instruments).

X-ray Crystallography

Suitable X-ray quality single crystals were grown in the glovebox over 2-3 days from concentrated (40-50 mM) solutions in diethyl ether kept at −30 °C. The solid-state structures of all the crystallized complexes show a hydrogen bonding interaction between *N*-atom of the protonated acids and O-atom of diethyl ether ($Et_2O\cdots H{-}N^{+}_{acid}$). The crystallographic asymmetric

unit in [4-MeMorphH][$B(C_6F_5)_4$]·Et_2O, [2,6-Me_2PyH][$B(C_6F_5)_4$]·Et_2O, and [$PhMe_2NH$][$B(C_6F_5)_4$]·Et_2O contains a diethyl ether molecule disordered over two orientations. The occupancy factor of the major component in diethyl ether disorder for [4-MeMorphH][$B(C_6F_5)_4$]·Et_2O refines to 0.682 while for [2,6-Me_2PyH]-[$B(C_6F_5)_4$]·Et_2O and [$PhMe_2NH$][$B(C_6F_5)_4$]·Et_2O, it refines to 0.595 and 0.771, respectively. The molecular packing for all the complexes is shown in Figures S31-S36. Meetsma *et al.*[a] and Osi *et al.*[b] reported the molecular structure of [$PhMe_2NH$][$B(C_6F_5)_4$] and [2,6-Me_2PyH][$B(C_6F_5)_4$], respectively, without any H-bonded diethyl ether in the crystal lattice and the average N–C bond distance as well as the C–N–C bond angle were similar to the H-bonded complex reported here.

All reflection intensities were measured at 100(2) K using a Gemini R diffractometer (equipped with Atlas detector) with CuKα radiation (λ = 1.54178 Å) under the program CrysAlisPro (Version CrysAlisPro 1.171.38.43f, Rigaku OD, 2015). The same program (but a different version viz. CrysAlisPro 1.171.40.53, Rigaku OD, 2019) was used to refine the cell dimensions and for data reduction. The structure was solved with the program SHELXT-2018/2 and was refined on F2 by full-matrix least-squares technique using the SHELXL-2018/3 program package.[c] Analytical absorption correction based on gaussian integration was applied using a multifaceted crystal model by CrysAlisPro. Non-hydrogen atoms were refined anisotropically. In the refinement, hydrogen atoms, except those involved in H-bonding, were treated as riding atoms using SHELXL default parameters while those involved in H-bonding were located with electron difference maps. For [4-MeMorpH][$B(C_6F_5)_4$]·Et_2O, the crystal lattice also contains a disordered diethyl ether molecule, for which its contributions have been taken out using the program SQUEEZE for the final refinement. All details of the SQUEEZE refinement are mentioned in the final CIF file.

Synthesis and Characterization

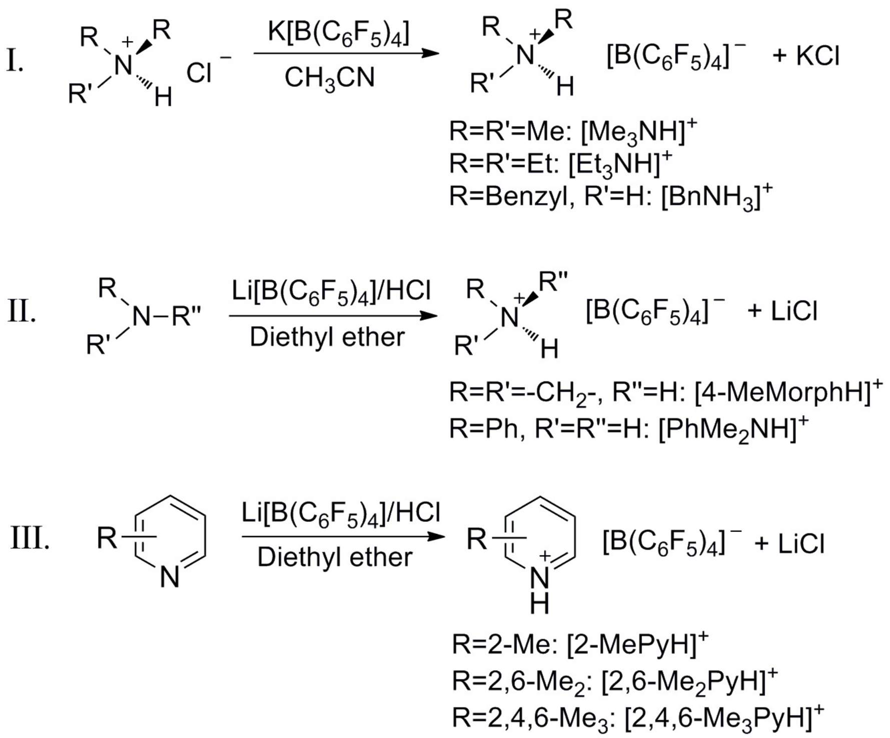

Scheme S1. Synthetic Schemes for the Preparation of the [$B(C_6F_5)_4$]$^-$ Salts of Protonated Nitrogen Species Described in This Study.

All of the $[B(C_6F_5)_4]^-$ salts of protonated nitrogen species were prepared through salt metathesis with $K[B(C_6F_5)_4]$ in MeCN or reaction of the nitrogen base with hydrochloric acid (HCl) in the presence of $Li[B(C_6F_5)_4]\cdot 2.35Et_2O$ in diethyl ether, which resulted in precipitation of KCl or LiCl, respectively, giving the desired products (Scheme S1). Preparation of the $[B(C_6F_5)_4]^-$ acids from the amine hydrochloride salts was performed by a general procedure; details are given for trimethylammonium $[B(C_6F_5)_4]^-$ as a representative case.

$[Me_3NH][B(C_6F_5)_4]\cdot 0.5Et_2O$

The trimethylamine hydrochloride salt was dried under vacuum before transferring into the glovebox. Into a 50 mL Schlenk flask was added a solution of $K[B(C_6F_5)_4]$ (487 mg, 676 mmol) in MeCN (12 mL). Then, a solution of the trimethylamine hydrochloride (64.7 mg, 676 mmol) in MeCN (11 mL) was added into the Schlenk flask. Upon combination of the two solutions, a fine, white precipitate was seen. The mixture was stirred for 30 minutes before filtration to remove the KCl. The resultant solution was then evaporated under reduced pressure and the white solid was redissolved in the minimum amount of Et_2O. The solution was kept in the freezer at –30°C. After 3 days, colorless crystals were observed suitable for X-ray structural determination. 87% Yield (410 mg, 0.5 Et_2O per acid molecule). FT-IR (solid) [cm^{-1}]: $\nu_{(N\text{-}H)}$ = 3357 (Figure S37). ^{1}H-NMR (DCM-d_2, 400 MHz; δ, ppm): 6.80 (t, $^1J_{NH}$ = 53.5 Hz, 1H); 3.03 (d, 9H) (Figure S2). ^{19}F-NMR (DCM-d_2, 376 MHz; δ, ppm): -133.21; -163.22; -167.27 (Figure S11).

$[Et_3NH][B(C_6F_5)_4]\cdot 0.8Et_2O$

Colorless crystals were collected from cold Et_2O, 346 mg (65%, 0.8 Et_2O per acid molecule). FT-IR (solid) [cm^{-1}]: $\nu_{(N\text{-}H)}$ = 3241 (Figure S38). ^{1}H-NMR (DCM-d_2, 400 MHz; δ, ppm): 5.44 (t, $^1J_{NH}$ = 52.9 Hz, 1H); 3.26 (dq, 6H); 1.38 (s, 9H) (Figure S1). ^{19}F-NMR (DCM-d_2, 376 MHz; δ, ppm): -133.14; -163.29; -167.31 (Figure S10).

$[BnNH_3][B(C_6F_5)_4]\cdot 2Et_2O$

White powder was collected, 427 mg (67%, 2 Et_2O per acid molecule and MeCN). FT-IR (solid) [cm^{-1}]: $\nu_{(N\text{-}H)}$= 3307 (Figure S39). ^{1}H-NMR (DCM-d_2, 400 MHz; δ, ppm): 6.96 (s, 3H); 7.49 (d, 3H); 7.35 (m, 2H); 4.20 (s, 2H) (Figure S3). ^{19}F-NMR (DCM-d_2, 376 MHz; δ, ppm): -133.22; -163.21; -167.23 (Figure S12). A broad singlet was observed for the N-H protons in ^{1}H-NMR due to the presence of a small amount of base in the starting compound (commercial HCl salt). After the addition of the strong acid $[H(OEt_2)_2][B(C_6F_5)_4]$ to our product, we were able to observe the triplet splitting of the N-H protons; (DCM-d_2, 500 MHz; δ, ppm): 6.33 (t, $^1J_{NH}$ = 50.6 Hz, 3H), see Figure S4.

Preparation of the $[B(C_6F_5)_4]^-$ acids from the conjugate base was performed by a general procedure; details are given for 2,4,6-trimethylpyridinium $[B(C_6F_5)_4]^-$ as a representative case.

$[2,4,6\text{-}Me_3PyH][B(C_6F_5)_4]\cdot 0.75Et_2O$

In the glovebox, a solution of $Li[B(C_6F_5)_4]$ (573 mg, 625 mmol, 1 eq.) in Et_2O (6 mL) was made in a Schlenk flask. This was transferred out of the glovebox and 2,4,6-collidine (83 µL, 625 mmol, 1 eq.) was added to the flask under argon. Then, the solution was cooled down with a cold bath (Dry ice, MeCN, –30°C). After cooling the solution, hydrogen chloride in ether (2 M, 2 mL, 6 eq.) was injected into the Schlenk flask. Upon swirling the solution, a white precipitate began to form. The solution was kept at –30°C for 30 minutes and swirled every 5 minutes. The solution was brought up to room temperature before the solvent was evaporated. In the glovebox, the precipitate was re-dissolved in

Et_2O and was filtered. The clear E_2O solution was then placed in the freezer at –30°C. After a few days, colorless crystals were observed suitable for X-ray structural determination. Yield (396 mg, 74%, 0.75 Et_2O per acid molecule). FT-IR (solid) [cm^{-1}]: $\nu_{(N-H)} = 3367$ (Figure S41). ^1H-NMR (DCM-d_2, 400 MHz; δ, ppm): 12.13 (t, $^1J_{NH}$ = 53.6 Hz, 1H); 7.40 (s, 2H); 2.71 (s, 6H); 2.58 (s, 3H) (Figure S6). ^{19}F-NMR (DCM-d_2, 376 MHz; δ, ppm): -133.08; -163.45; -167.39 (Figure S14).

[2,6-Me$_2$PyH][B(C$_6$F$_5$)$_4$]·0.5Et$_2$O

White powder was collected, 398 mg (78%, 0.5 Et_2O per acid molecule). FT-IR (solid) [cm^{-1}]: $\nu_{(N-H)} =$ 3308 (Figure S42). ^1H-NMR (DCM-d_2, 400 MHz; δ, ppm): 12.45 (t, $^1J_{NH}$ = 61.3 Hz, 1H); 8.33 (t, 3H); 7.65 (d, 2H); 2.81 (s, 6H) (Figure S7). ^{19}F-NMR (DCM-d_2, 376 MHz; δ, ppm): -133.08; -163.41; -167.37 (Figure S15).

[2-MePyH][B(C$_6$F$_5$)$_4$]·0.5Et$_2$O

White powder was collected, 438 mg (83%, 0.5 Et_2O per acid molecule). FT-IR (solid) [cm^{-1}]: $\nu_{(N-H)} =$ 3358 (Figure S43). ^1H-NMR (DCM-d_2, 400 MHz; δ, ppm): 12.68 (t, $^1J_{NH}$ = 65.1 Hz, 1H); 8.53 (td, 1H); 8.46 (t, 1H); 7.92 (m, 2H); 2.87 (s,3H) (Figure 8). ^{19}F-NMR (DCM-d_2, 376 MHz; δ, ppm): -133.12; -163.33; -167.30 (Figure S16).

[4-MeMorphH][B(C$_6$F$_5$)$_4$]·0.3Et$_2$O

Colorless crystals were collected, 358 mg (70%, 0.3 Et_2O per acid molecule). FT-IR (solid) [cm^{-1}]: $\nu_{(N-H)} = 3239$ (Figure S40). ^1H-NMR (DCM-d_2, 400 MHz; δ, ppm): 6.50 (t, $^1J_{NH}$ = 53.2 Hz, 1H); 4.20 (dd, J = 13.9, 3.6 Hz, 2H$_d$); 3.73 (ddd, J = 13.9, 11.6, 2.7 Hz, 2H$_c$); 3.49 (m, 2H$_b$); 3.22 (dddd, J = 12.5, 11.6, 3.6, 2.5 Hz, 2H$_a$); 3.03 (d, 3H) (Figure S5). ^{19}F-NMR (DCM-d_2, 376 MHz; δ, ppm): -133.21; -163.05; -167.13 (Figure S13). ^1H-NMR (DCB-d_4, 400 MHz; δ, ppm): 6.59 (br, 1H); 3.73 (m, 2H$_d$); 3.31 (m, 2H$_c$); 2.99 (m, 2H$_b$); 2.66 (m, 2H$_a$); 2.57 (s, 3H) (Figure S30).

[PhMe$_2$NH][B(C$_6$F$_5$)$_4$]·0.7Et$_2$O

Colorless crystals were collected, 331 mg (62%, 0.7 Et_2O per acid molecule). FT-IR (solid) [cm^{-1}]: $\nu_{(N-H)} = 3251$ (Figure S44). ^1H-NMR (DCM-d_2, 400 MHz; δ, ppm): 8.81 (t, $^1J_{NH}$ = 42.0 Hz, 1H); 7.64 (m, 3H); 7.41 (m, 2H); 3.35 (s, 6H) (Figure S9). ^{19}F-NMR (DCM-d_2, 376 MHz; δ, ppm): -133.14; -163.26; -167.27 (Figure S17).

The resulting $[B(C_6F_5)_4]^-$ salts show a markedly increased solubility in a broad range of organic solvents including the more polar acetonitrile (MeCN) and acetone but also lower polarity solvents such as dichloromethane (DCM), tetrahydrofuran (THF), and diethyl ether (Et_2O).

[Et$_3$NH][SbF$_6$]

The $[SbF_6]^-$ salt of triethylamine was prepared through salt metathesis in MeCN. First, the triethylamine hydrochloride salt was dried under vacuum before transferring it into the glovebox. Into a 50 mL Schlenk flask, a solution of triethylamine hydrochloride (69 mg, 500 µmol) in MeCN (12 mL) was added. Then, a solution of Ag[SbF_6] (172 mg, 500 µmol) in MeCN (8 mL) was added to the Schlenk flask. After addition of the Ag[SbF_6] solution, a fine white cloud was observed. The mixture was stirred for 40 minutes before filtering off the AgCl precipitate and the resultant solution was evaporated under vacuum to produce a white solid (143 mg, 85%). FT-IR (solid) [cm^{-1}]: $\nu_{(SbF6)} = 641$ and 651 and $\nu_{(N-H)} = 3200$ (Figure S45). ^1H-NMR (NB-d_5, 400 MHz; δ, ppm): 6.68 (t, 1H); 3.59 (m, 6H); 1.57 (t, 9H) (Figure S19). ^{19}F-NMR (NB-d_5, 376 MHz; δ, ppm): -122.5 (Figure S20).

NMR Spectroscopy

Chemical shifts were referenced against solvent residual shifts (e.g., DCM-d_2 at δ = 5.32 ppm) and/or tetramethylsilane (TMS at δ = 0.00 ppm). Diethyl ether shifts were found in the proton NMR spectrum of each protonated amine (at δ = 1.12-1.21 ppm and δ = 3.46-3.62 ppm).

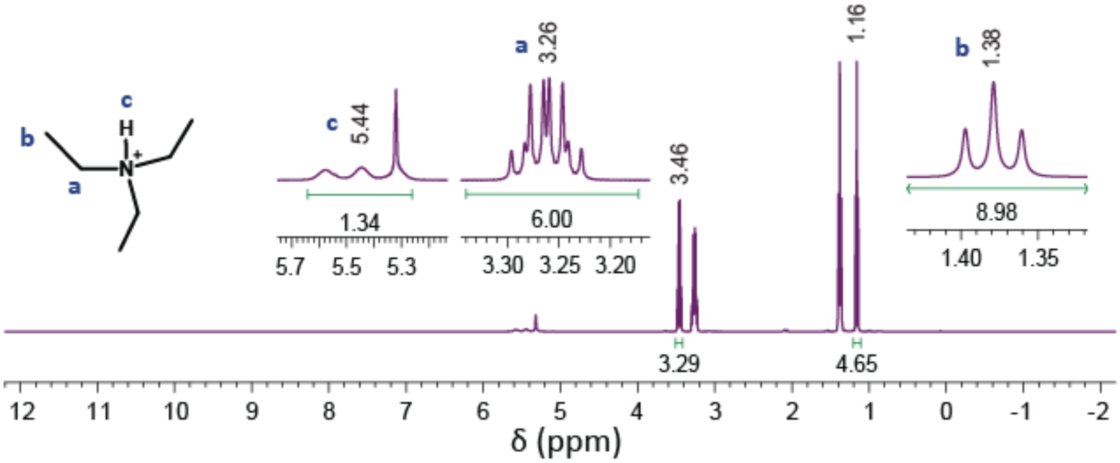

Figure S1. ^{1}H-NMR spectrum of $[Et_3NH][B(C_6F_5)_4]$ in DCM-d_2 (400MHz) at room temperature.

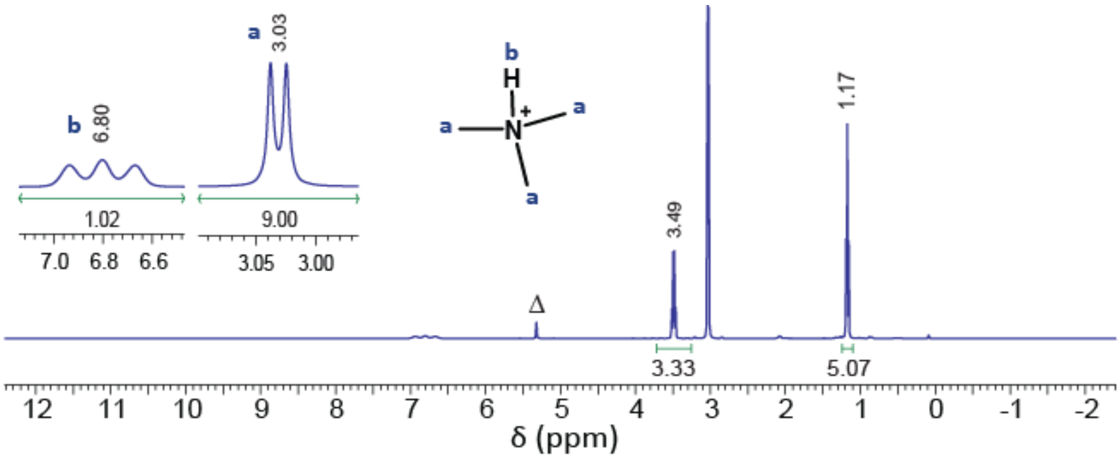

Figure S2. ^{1}H-NMR spectrum of $[Me_3NH][B(C_6F_5)_4]$ in DCM-d_2 (400 MHz) at room temperature.

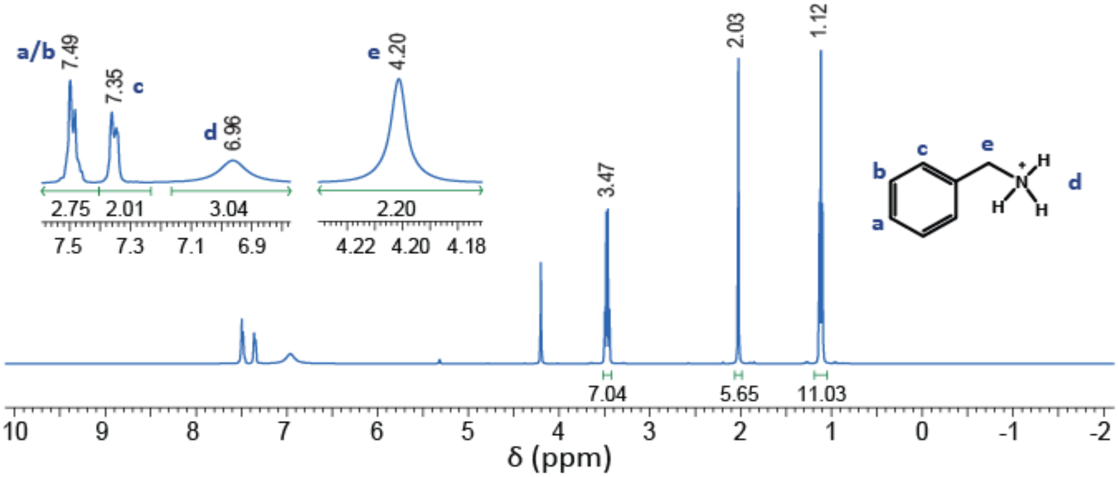

Figure S3. ^{1}H-NMR spectrum of $[BnNH_3][B(C_6F_5)_4]$ in DCM-d_2 (400 MHz) at room temperature.

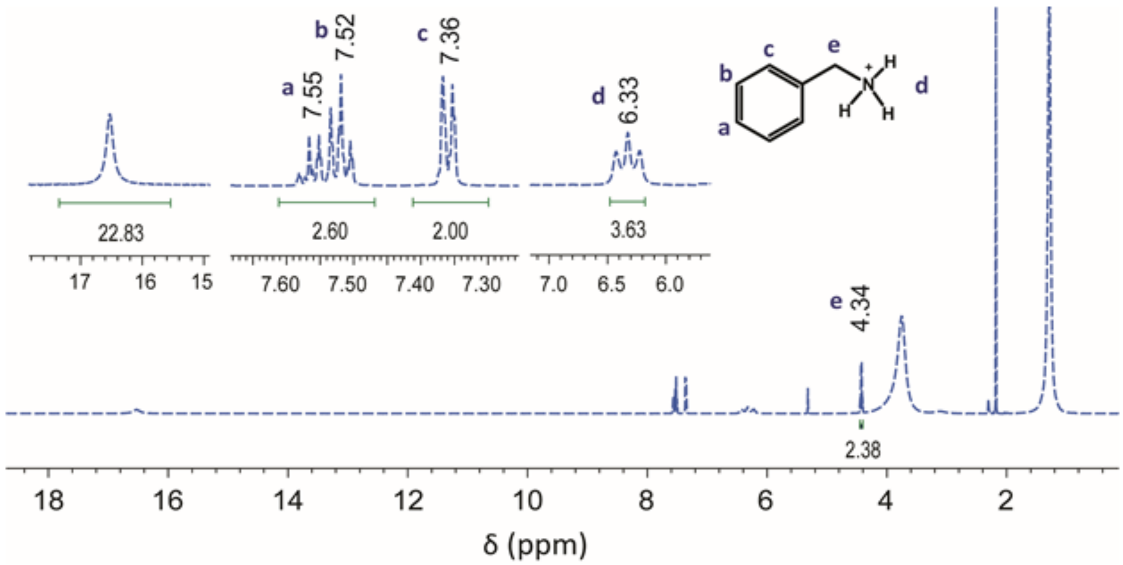

Figure S4. ^{1}H-NMR spectrum of $[BnNH_3][B(C_6F_5)_4]$ with 4 equivalents of $[H(OEt_2)_2][B(C_6F_5)_4]$ in DCM-d_2 (500 MHz)at room temperature.

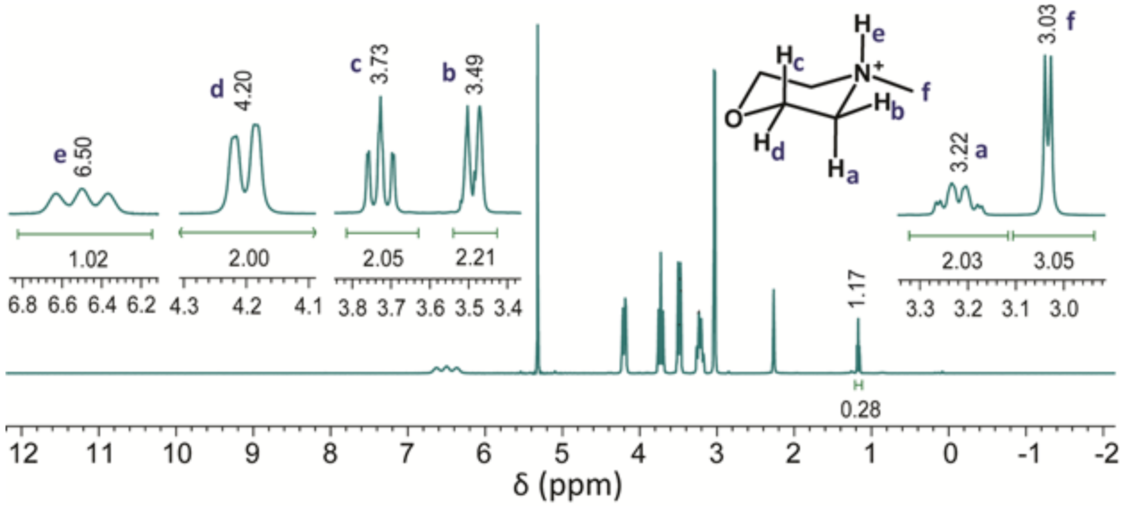

Figure S5. ^{1}H-NMR spectrum of $[4\text{-}MeMorphH][B(C_6F_5)_4]$ in DCM-d_2 (400 MHz) at room temperature.

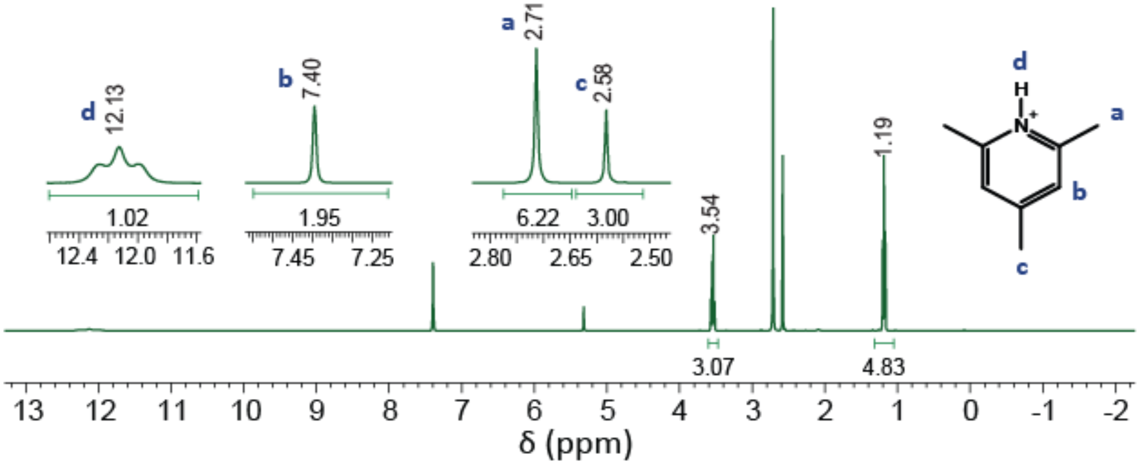

Figure S6. ^{1}H-NMR spectrum of $[2,4,6\text{-}Me_3PyH][B(C_6F_5)_4]$ in DCM-d_2 (400 MHz) at room temperature.

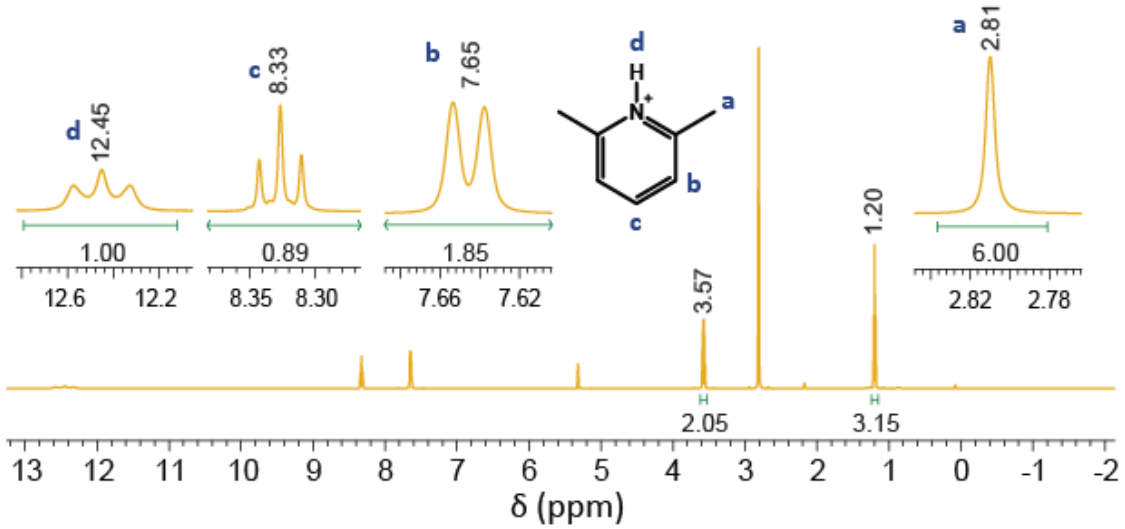

Figure S7. ^{1}H-NMR spectrum of $[2,6\text{-}Me_2PyH][B(C_6F_5)_4]$ in DCM-d_2 (400 MHz) at room temperature.

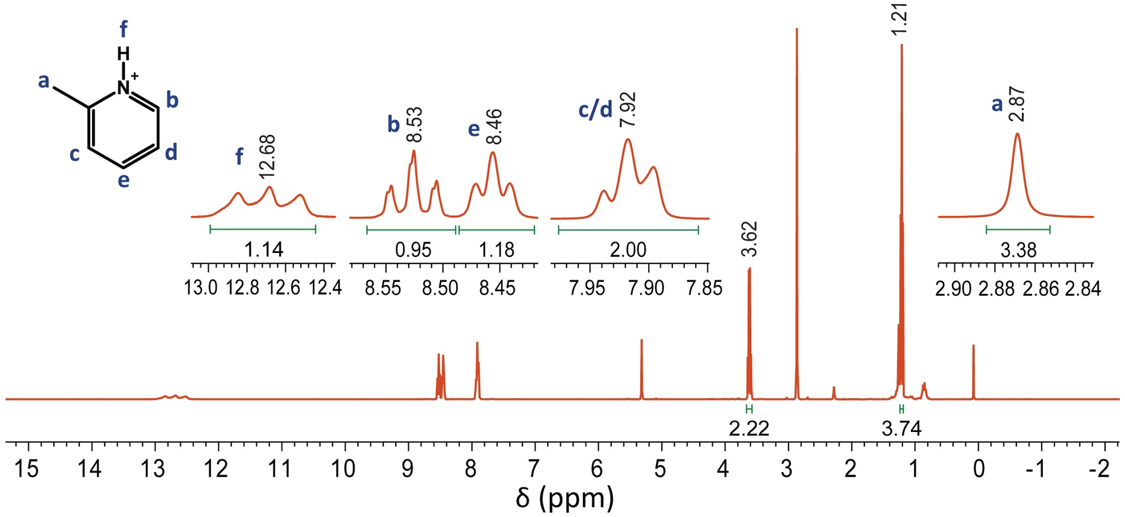

Figure S8. ^{1}H-NMR spectrum of $[2\text{-}MePyH][B(C_6F_5)_4]$ in DCM-d_2 (400 MHz) at room temperature.

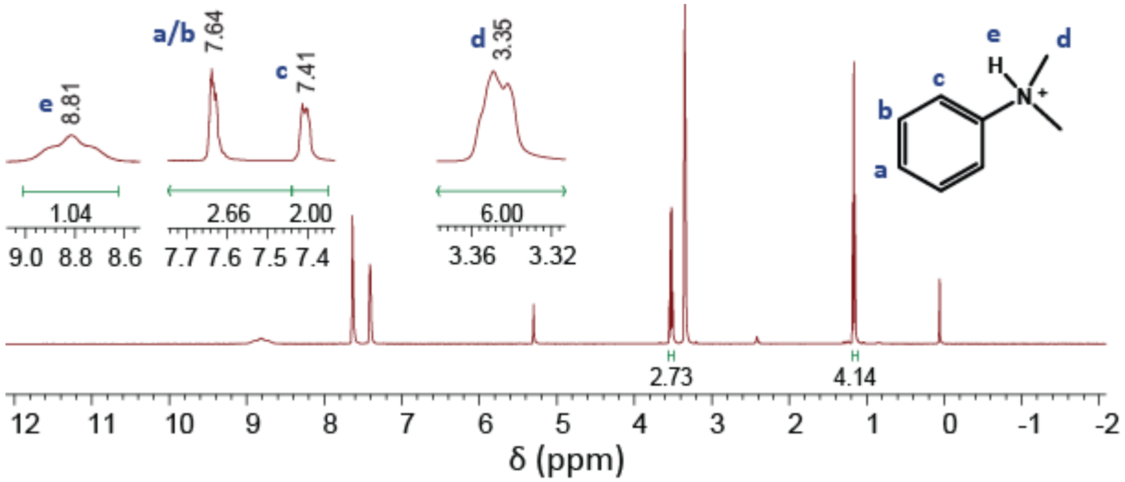

Figure S9. ^{1}H-NMR spectrum of $[PhMe_2NH][B(C_6F_5)_4]$ in DCM-d_2 (400 MHz) at room temperature.

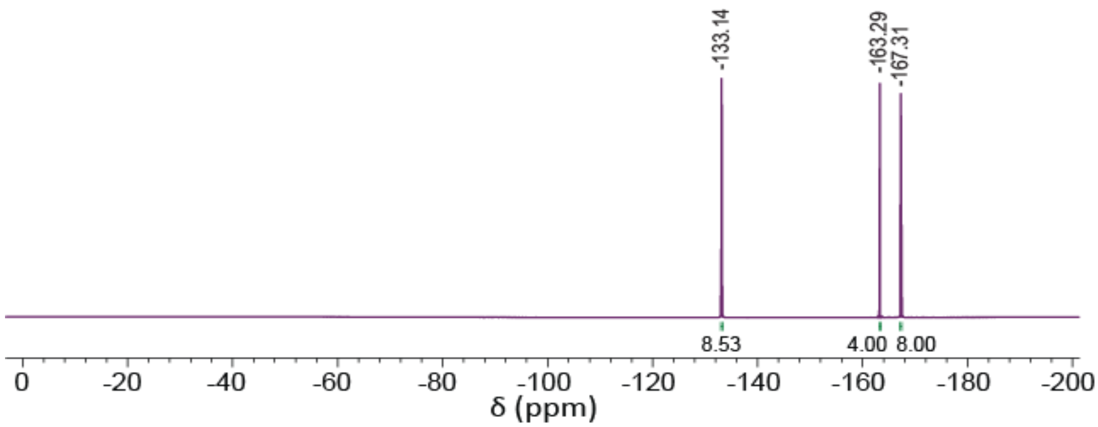

Figure S10. ^{19}F-NMR spectrum of $[Et_3NH][B(C_6F_5)_4]$ in DCM-d_2 (376 MHz) at room temperature.

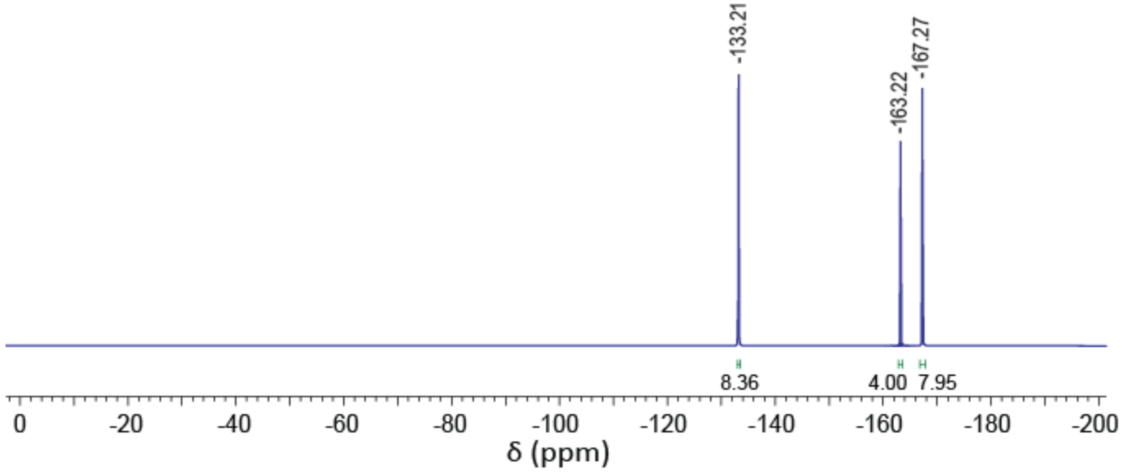

Figure S11. ^{19}F-NMR spectrum of $[Me_3NH][B(C_6F_5)_4]$ in DCM-d_2 (376 MHz) at room temperature.

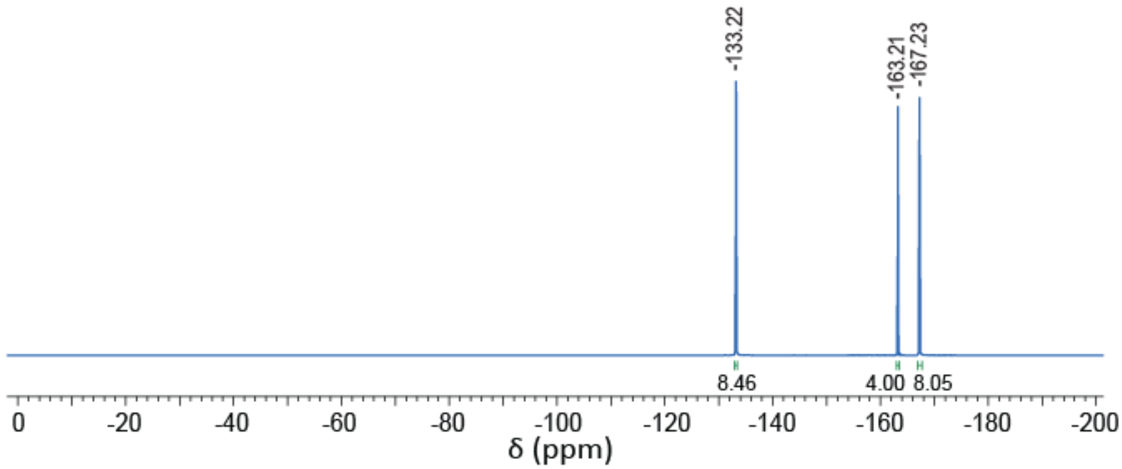

Figure S12. ^{19}F-NMR spectrum of $[BnNH_3][B(C_6F_5)_4]$ in DCM-d_2 (376 MHz) at room temperature.

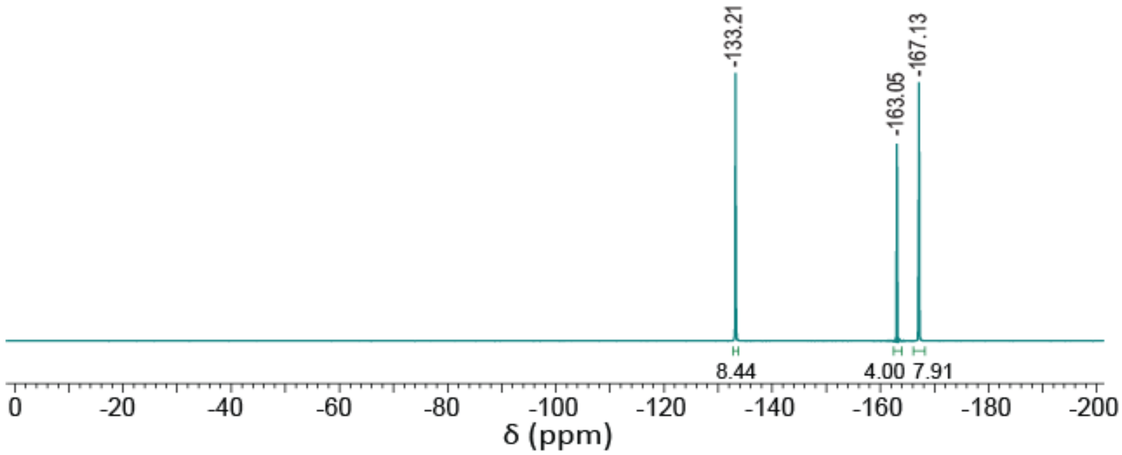

Figure S13. ^{19}F-NMR spectrum of [4-MeMorphH][$B(C_6F_5)_4$] in DCM-d_2 (376 MHz) at room temperature.

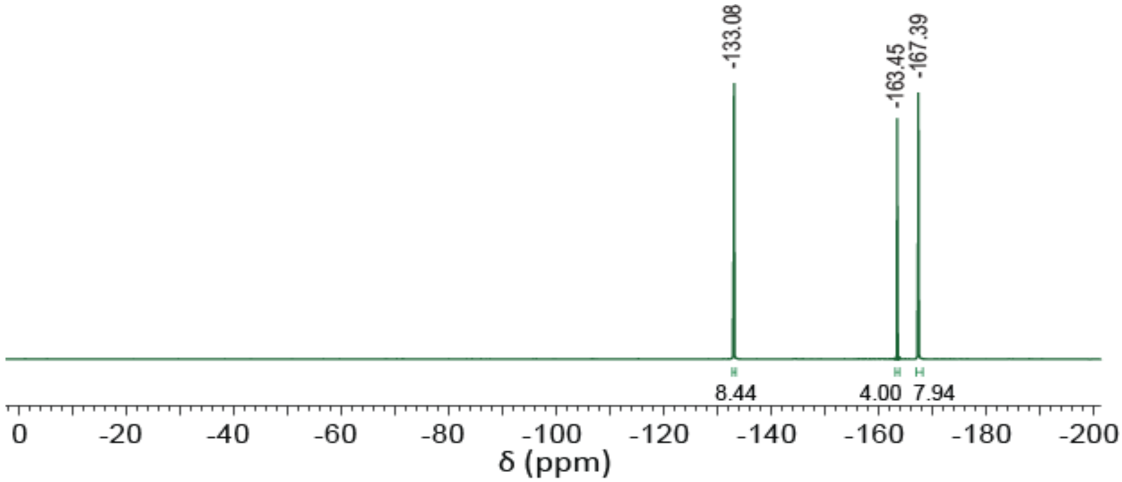

Figure S14. ^{19}F-NMR spectrum of [2,4,6-Me_3PyH][$B(C_6F_5)_4$] in DCM-d_2 (376 MHz) at room temperature.

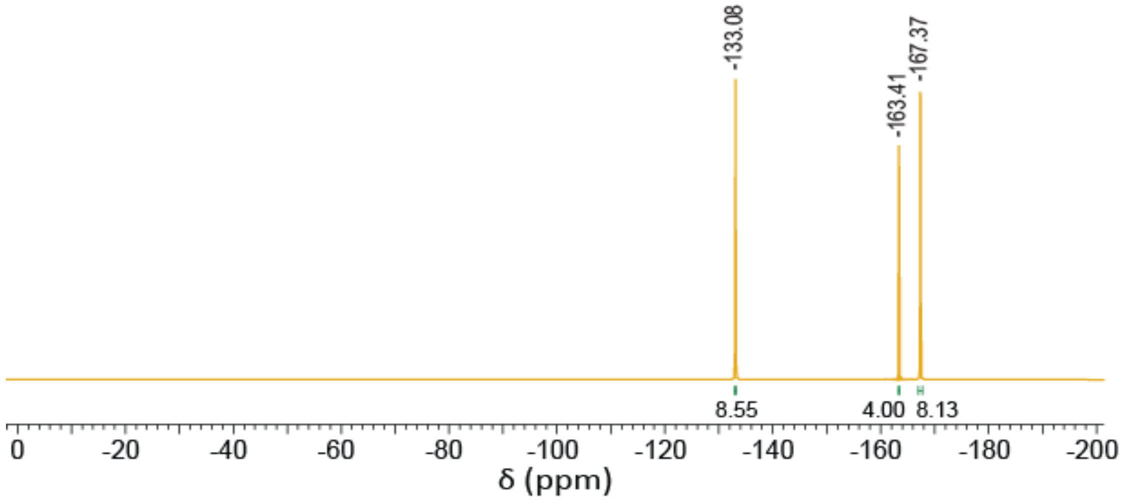

Figure S15. ^{19}F-NMR spectrum of [2,6-Me_2PyH][$B(C_6F_5)_4$] in DCM-d_2 (376 MHz) at room temperature.

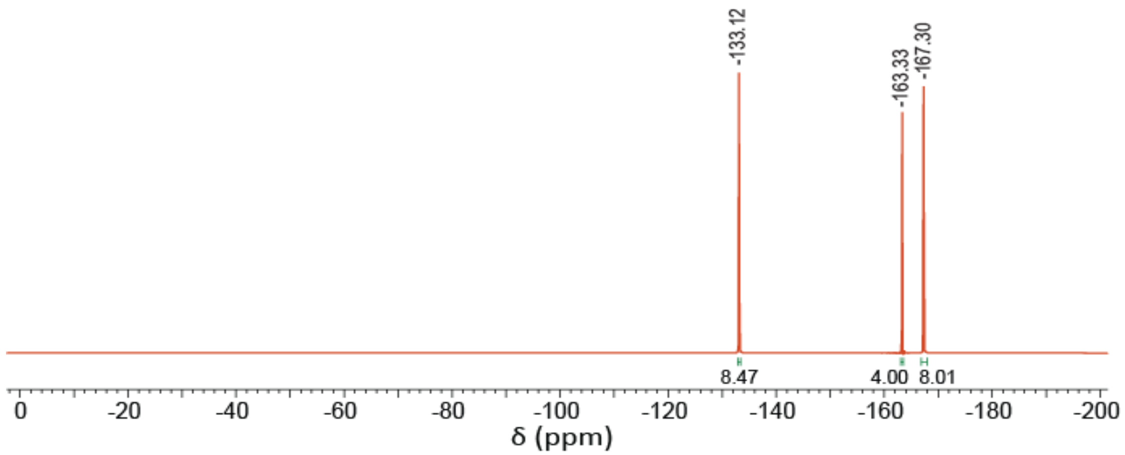

Figure S16. ^{19}F-NMR spectrum of [2-MePyH][B(C_6F_5)$_4$] in DCM-d_2 (376 MHz) at room temperature.

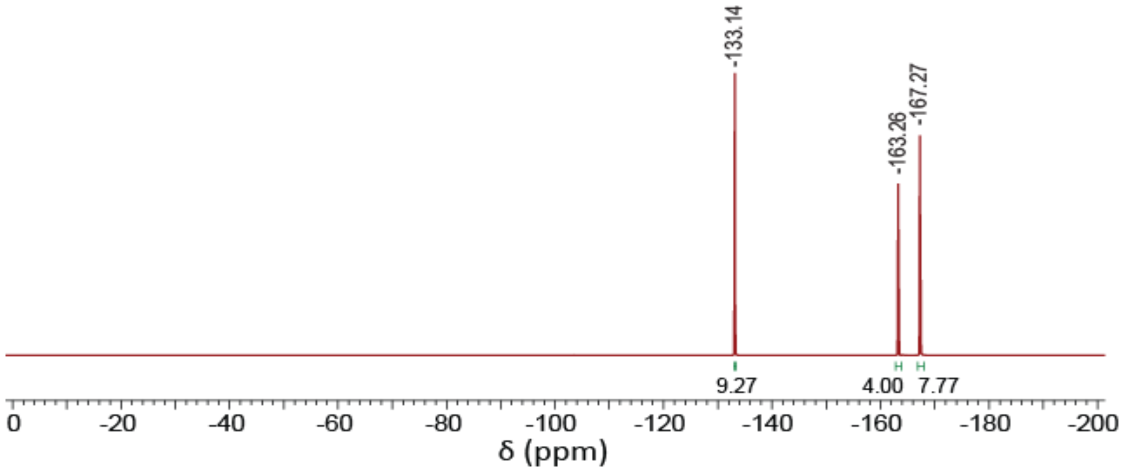

Figure S17. ^{19}F-NMR spectrum of [$PhMe_2NH$][B(C_6F_5)$_4$] in DCM-d_2 (376 MHz) at room temperature.

Table S1. Coupling constants ($^1J_{NH}$) of the ^{14}N-^{1}H triplets for the [B(C_6F_5)$_4$]$^-$ salts of protonated amines.

Protonated amine	$^1J_{NH}$ (average, Hz)	NMR Magnet Strength (MHz)
N,N-Dimethylanilinium	41.96	400
2-Methylpyridinium	65.06	400
2,6-Dimethylpyridinium	61.29	400
2,4,6-Trimethylpyridinium	53.55	400
4-methylmorpholinium	53.16	400
Benzylammonium	50.60	500
Trimethylaminium	53.52	400
Triethylaminium	52.86	400

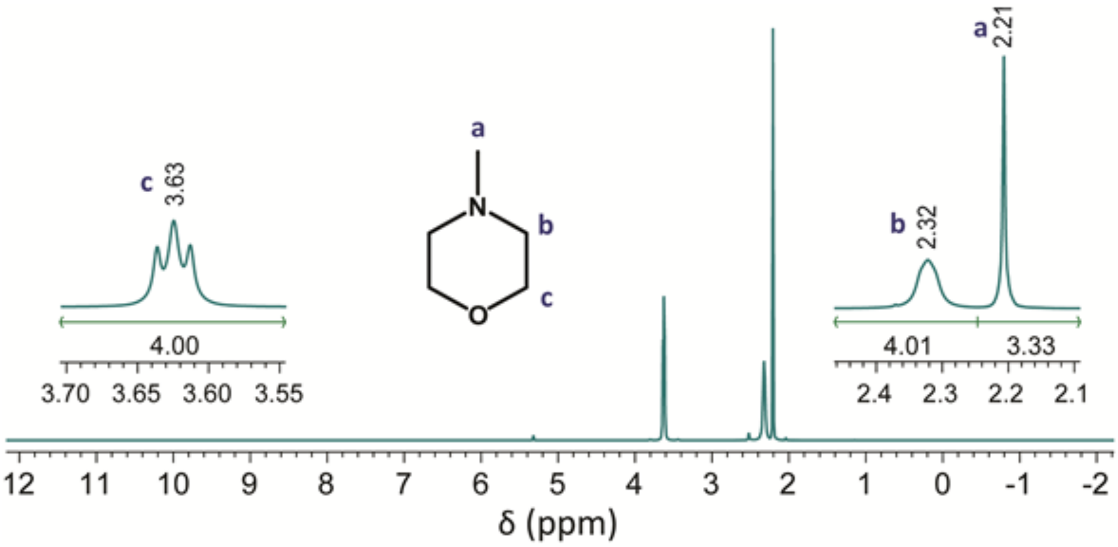

Figure S18. ^{1}H-NMR spectrum of 4-MeMorph in DCM-d_2 (500 MHz) at room temperature.

^{1}H-NMR Measurements in Various Solvents

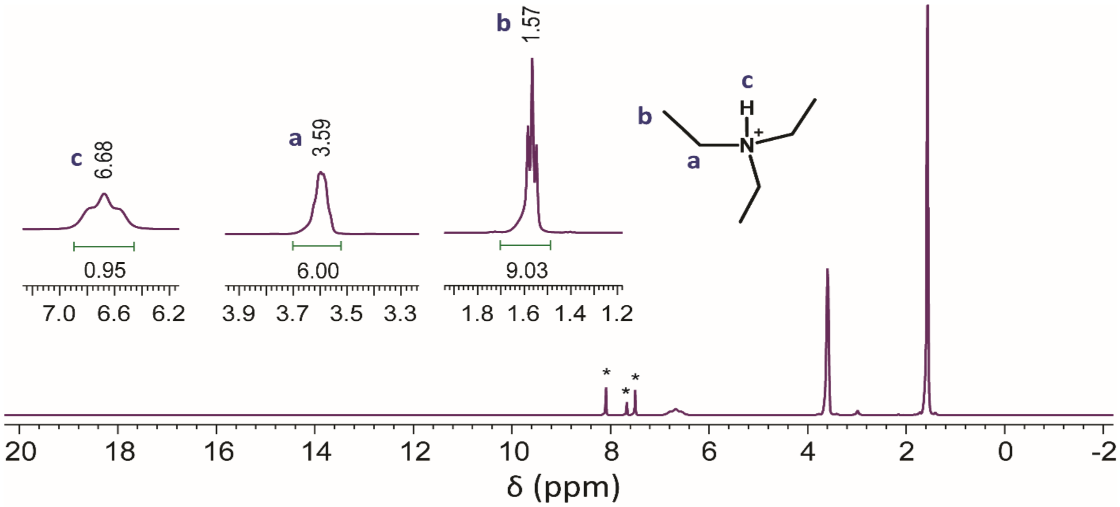

Figure S19. ^{1}H-NMR spectrum of $[Et_3NH][SbF_6]$ in NB-d_5 (400MHz) at room temperature.

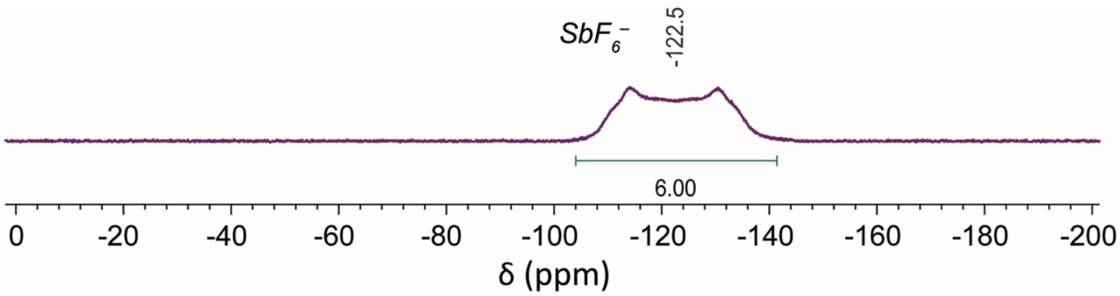

Figure S20. ^{19}F-NMR spectrum of $[Et_3NH][SbF_6]$ in NB-d_5 (376 MHz) at room temperature.

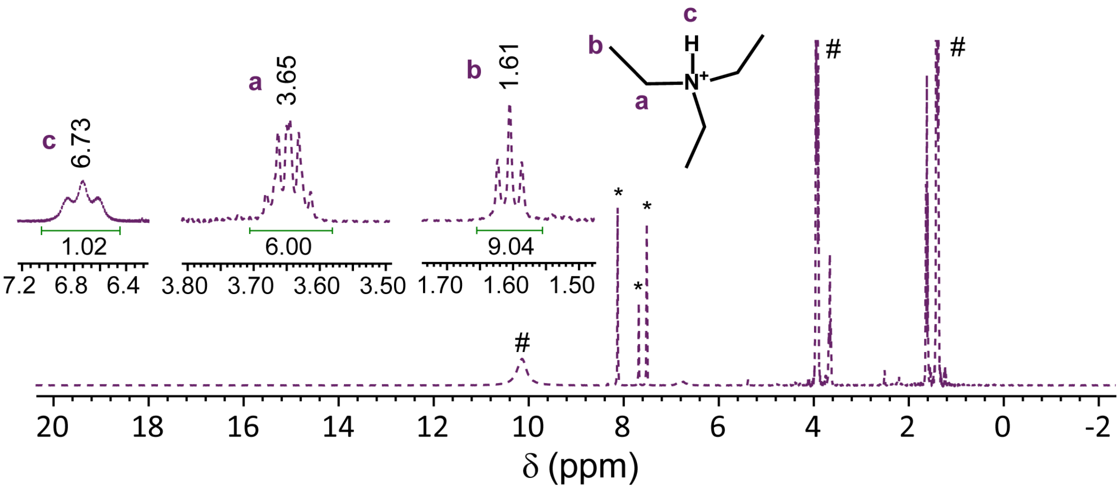

Figure S21. ^{1}H-NMR spectrum of of $[Et_3NH][B(C_6F_5)_4]$ with excess of $[H(OEt_2)_2][B(C_6F_5)_4]$ in NB-d_5 (400 MHz) at room temperature. Peaks labeled as '#' are from $[H(OEt_2)_2][B(C_6F_5)_4]$.

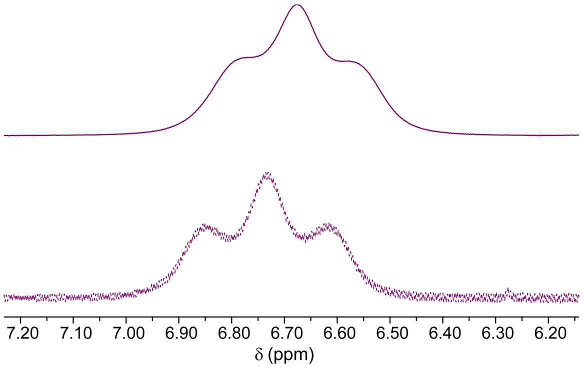

Figure S22. Part of the ^{1}H-NMR spectra of $[Et_3NH][SbF_6]$ (solid line) and $[Et_3NH][B(C_6F_5)_4]$ (dashed line) in NB-d_5 (400 MHz) at room temperature displaying the more significant broadening of the acidic proton signal due to the specific and stronger ion-pairing interaction of $[SbF_6]^-$ as compared to that of $[B(C_6F_5)_4]^-$.

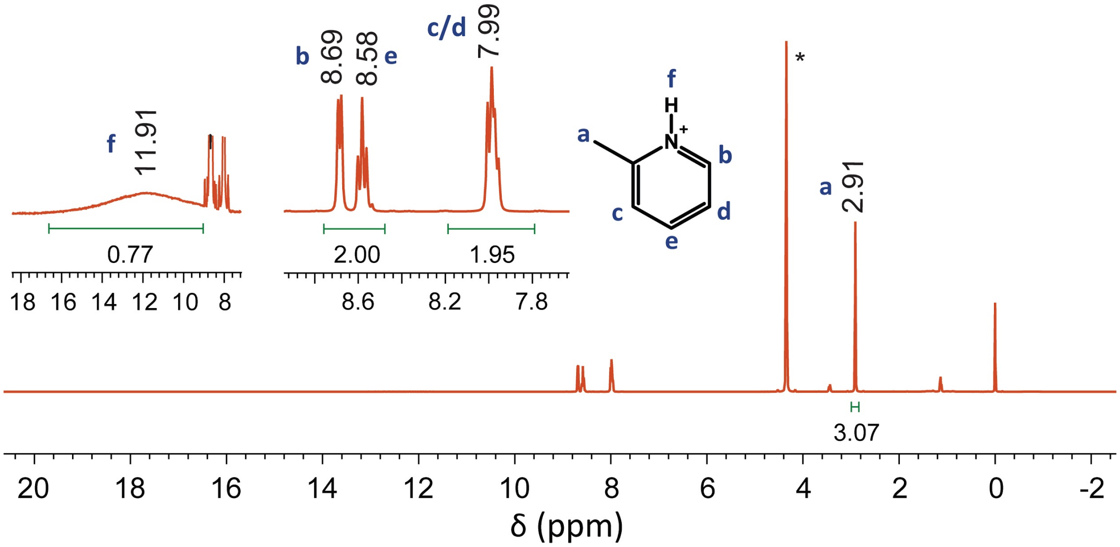

Figure S23. ^{1}H-NMR spectrum of $[2\text{-MePyH}][B(C_6F_5)_4]\cdot Et_2O$ in nitromethane-d_3 (400 MHz) at room temperature.

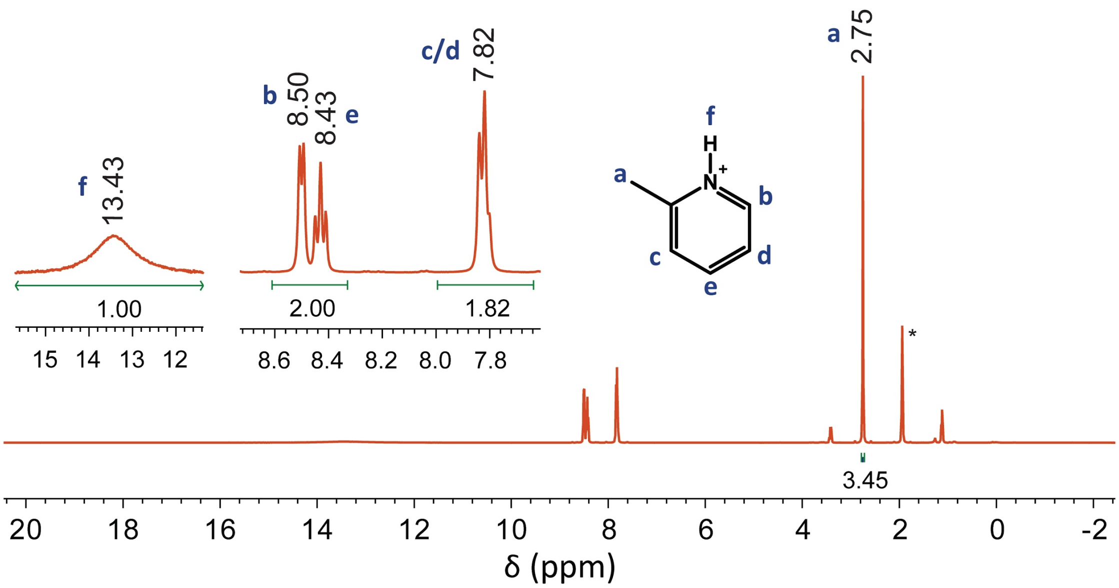

Figure S24. ^{1}H-NMR spectrum of [2-MePyH][B(C_6F_5)$_4$]·Et_2O in MeCN-d_3 (400 MHz) at room temperature.

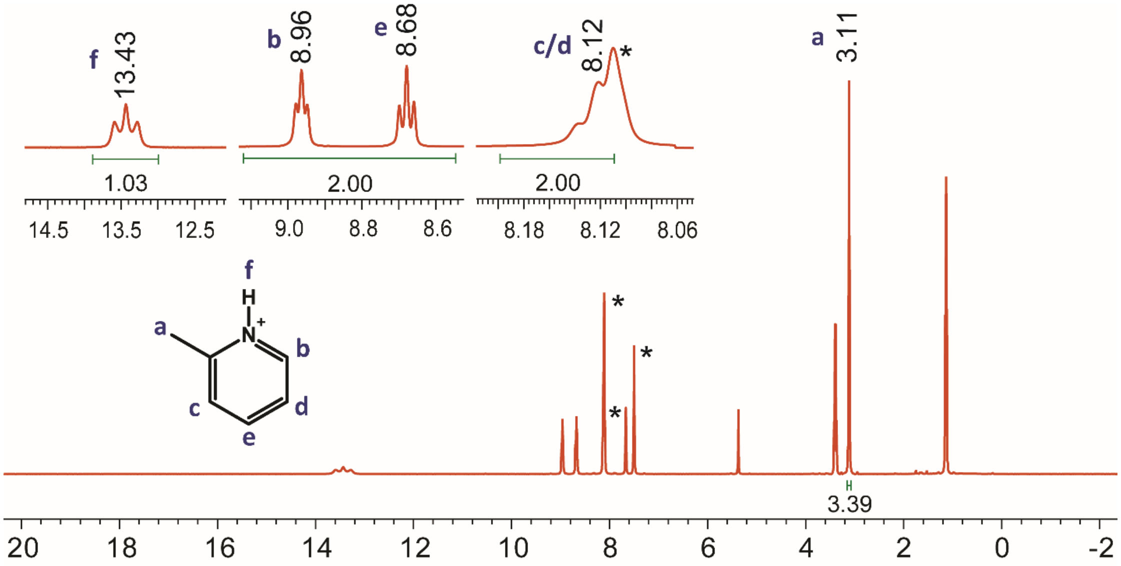

Figure S25. ^{1}H-NMR spectrum of [2-MePyH][B(C_6F_5)$_4$]·Et_2O in nitrobenzene-d_5 (400 MHz) at room temperature.

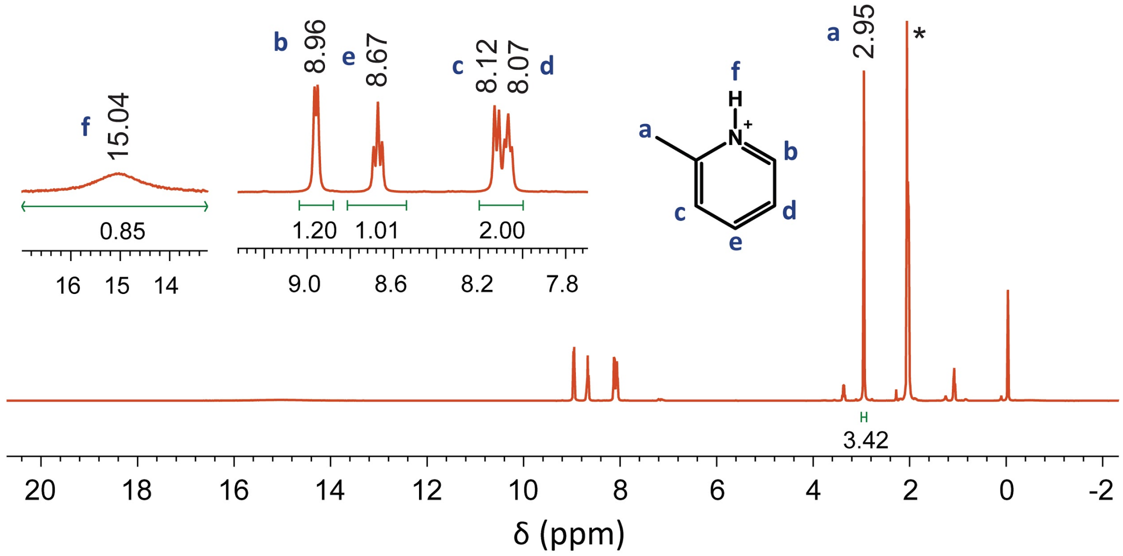

Figure S26. ^{1}H-NMR spectrum of $[2\text{-MePyH}][B(C_6F_5)_4]\cdot Et_2O$ in acetone-d_6 (400 MHz) at room temperature.

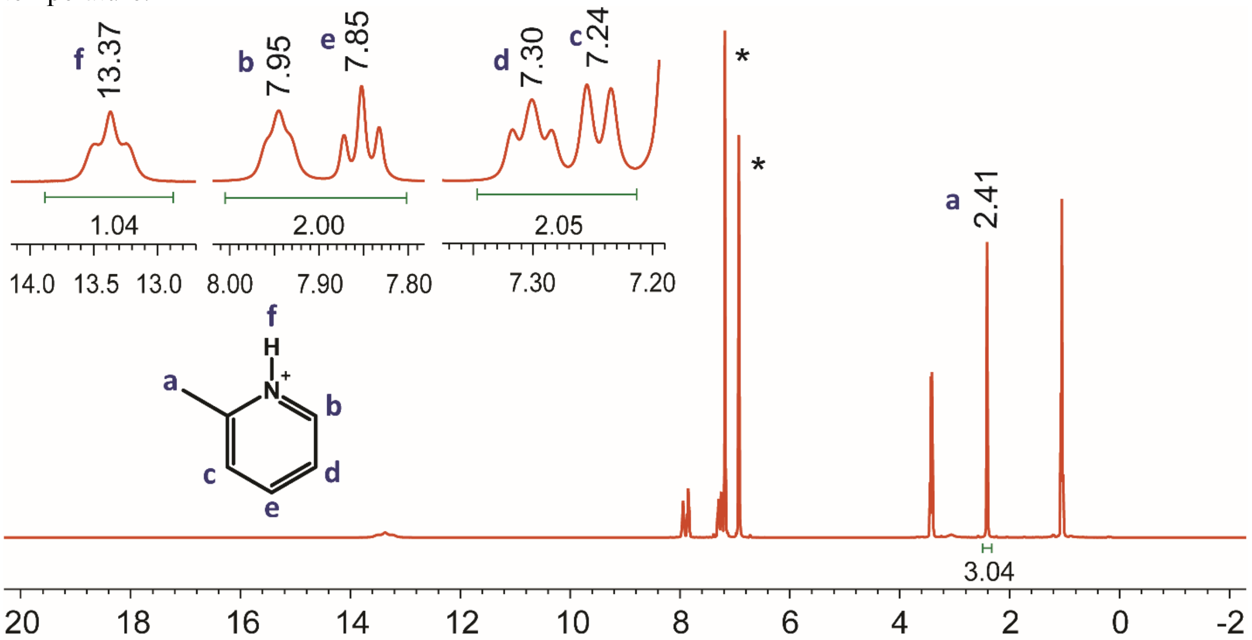

Figure S27. ^{1}H-NMR spectrum of $[2\text{-MePyH}][B(C_6F_5)_4]\cdot Et_2O$ in 1,2-dichlorobenzne-d_4 (400 MHz) at room temperature.

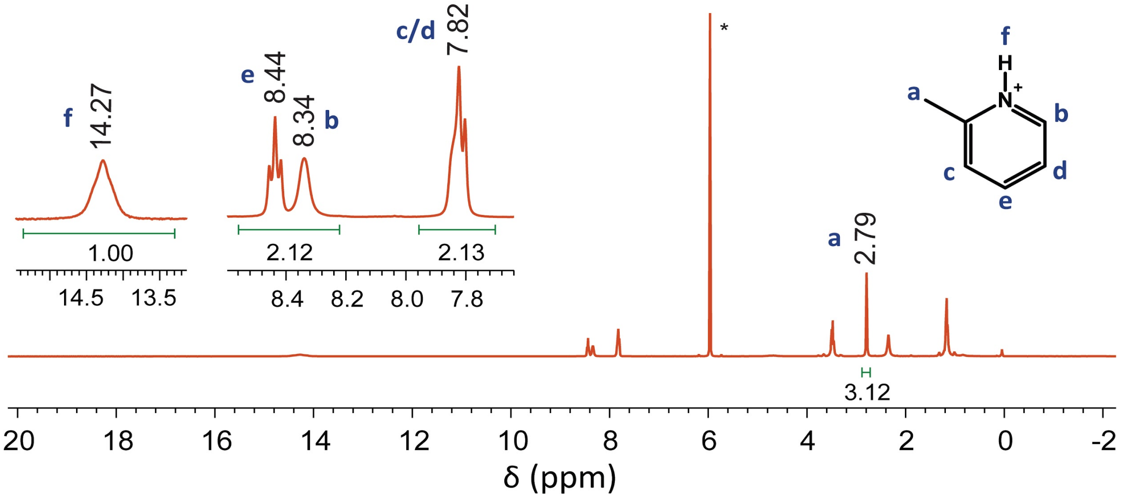

Figure S28. ^{1}H-NMR spectrum of [2-MePyH][B(C_6F_5)$_4$]·Et_2O in 1,1,2,2-tetrachloroethane-d_2 (400 MHz) at room temperature.

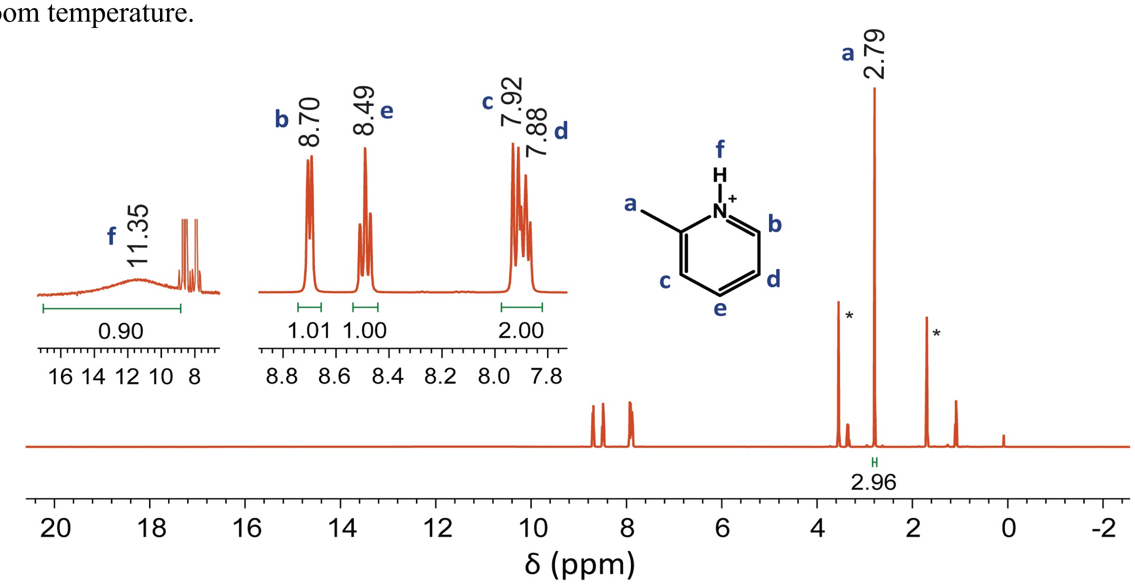

Figure S29. ^{1}H-NMR spectrum of [2-MePyH][B(C_6F_5)$_4$]·Et_2O in THF-d_8 (400 MHz) at room temperature.

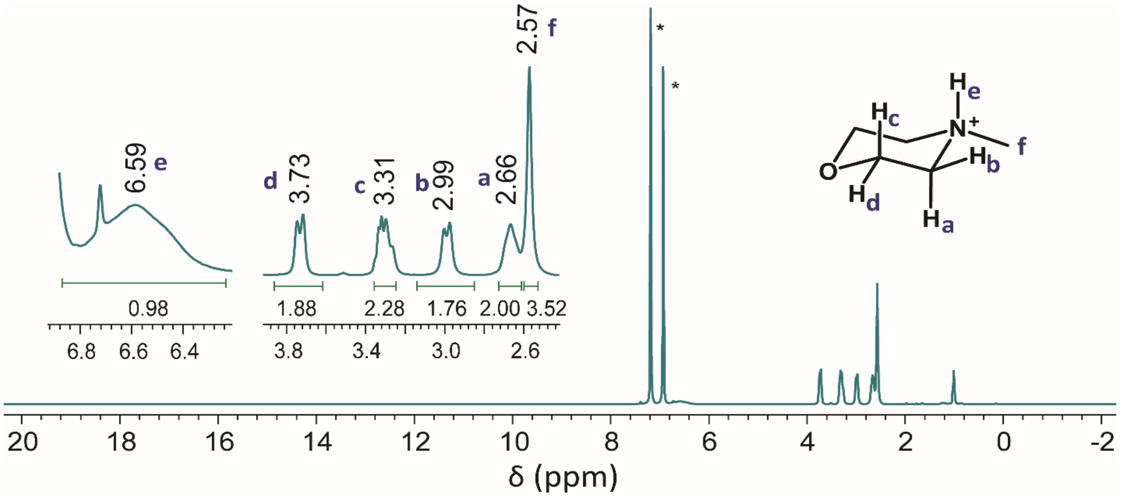

Figure S30. ^{1}H-NMR spectrum of [4-MeMorphH][B(C_6F_5)$_4$] in DCB-d_4 (400 MHz) at room temperature.

Crystallographic Studies

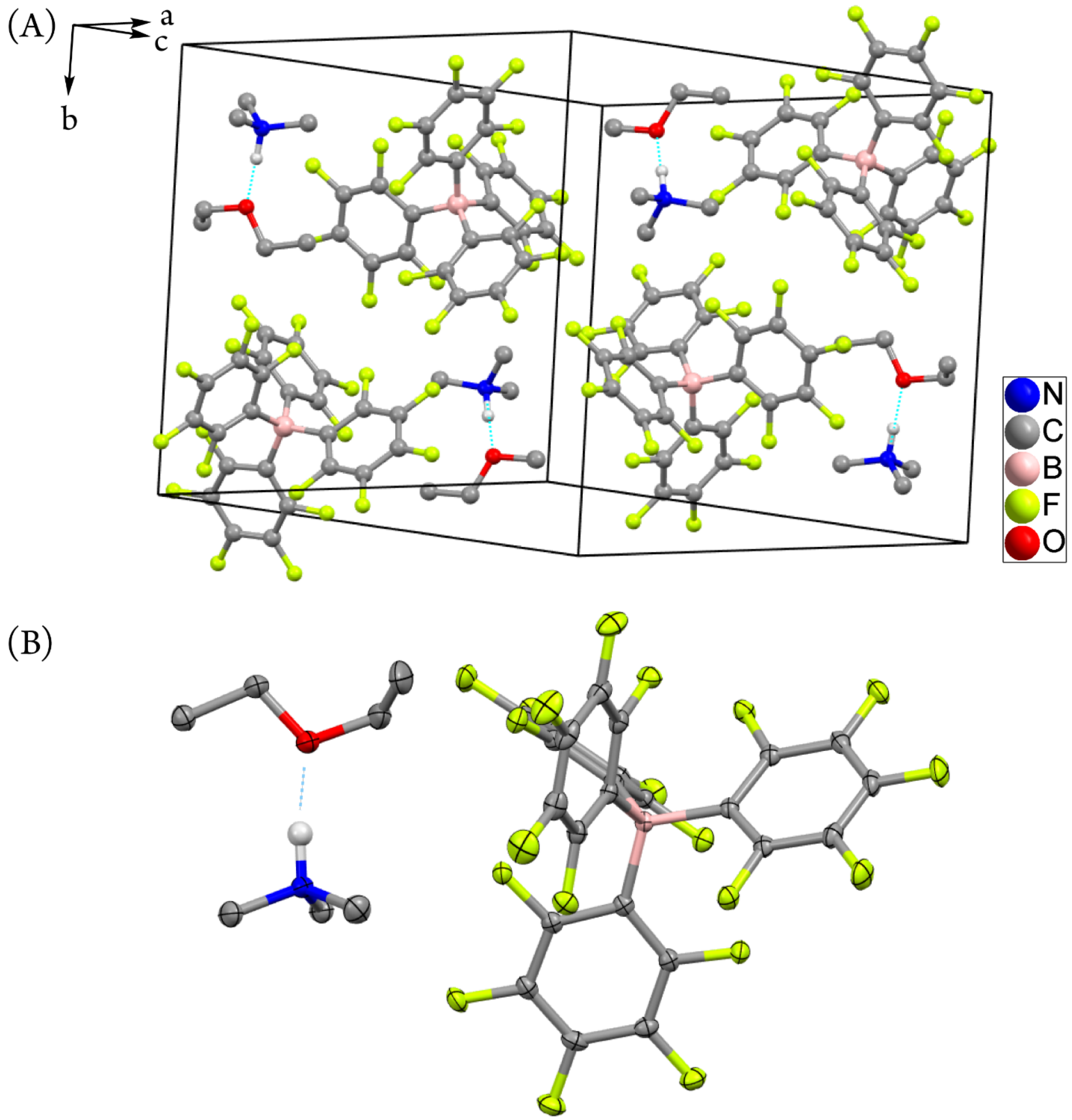

Figure S31. Diagram illustrating the (A) molecular packing and (B) displacement ellipsoid plot (50% probability level) of $[Me_3NH][B(C_6F_5)_4]\cdot Et_2O$ at 100(2) K. All of the hydrogens except for the acidic proton have been omitted for clarity. Dotted lines show the H-bonding interactions between the protonated nitrogen species and diethyl ether molecules.

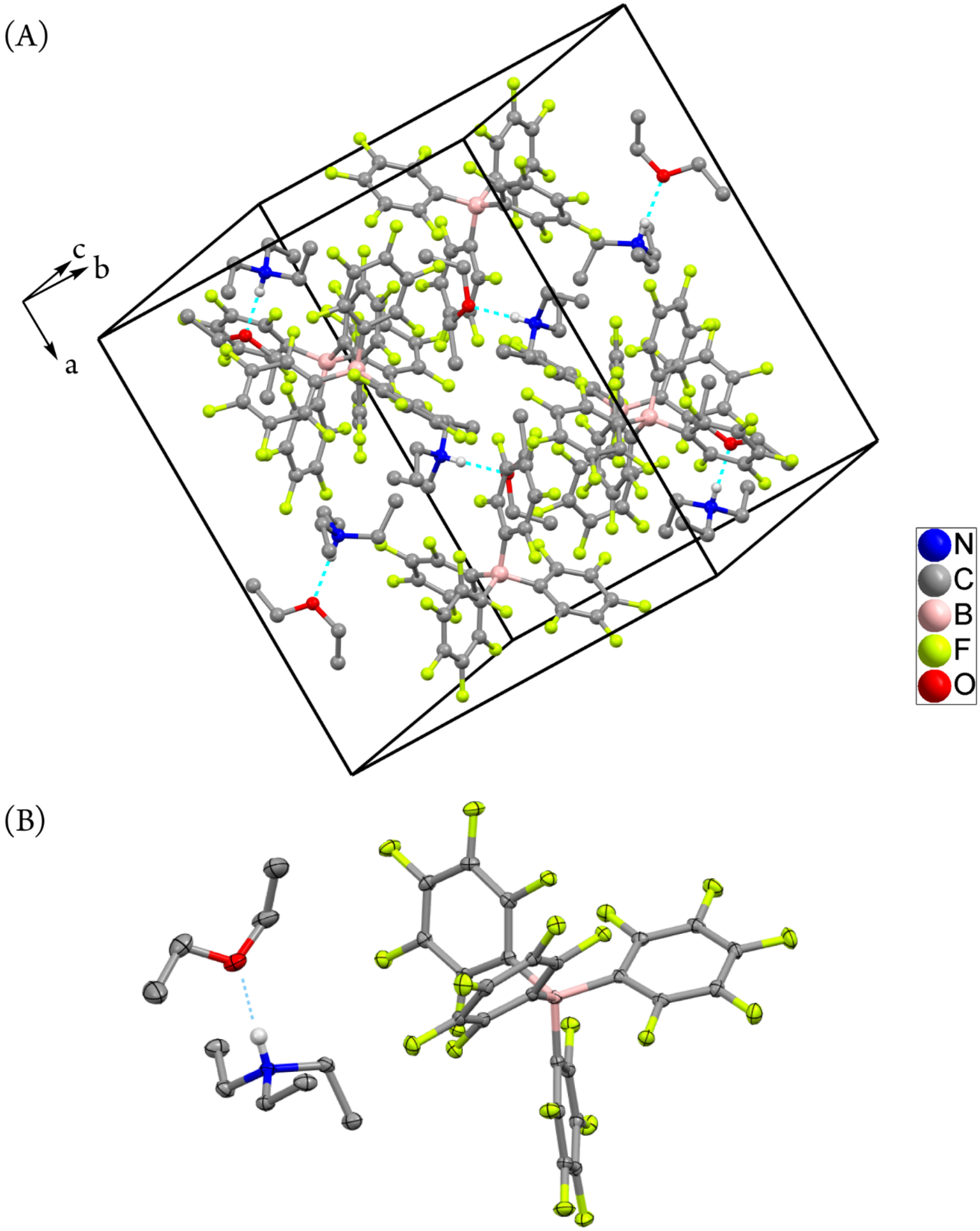

Figure S32. Diagram illustrating the (A) molecular packing and (B) displacement ellipsoid plot (50% probability level) of $[Et_3NH][B(C_6F_5)_4]\cdot Et_2O$ at 100(2) K. All of the hydrogens except for the acidic proton have been omitted for clarity. Dotted lines show the H-bonding interactions between the protonated nitrogen species and diethyl ether molecules.

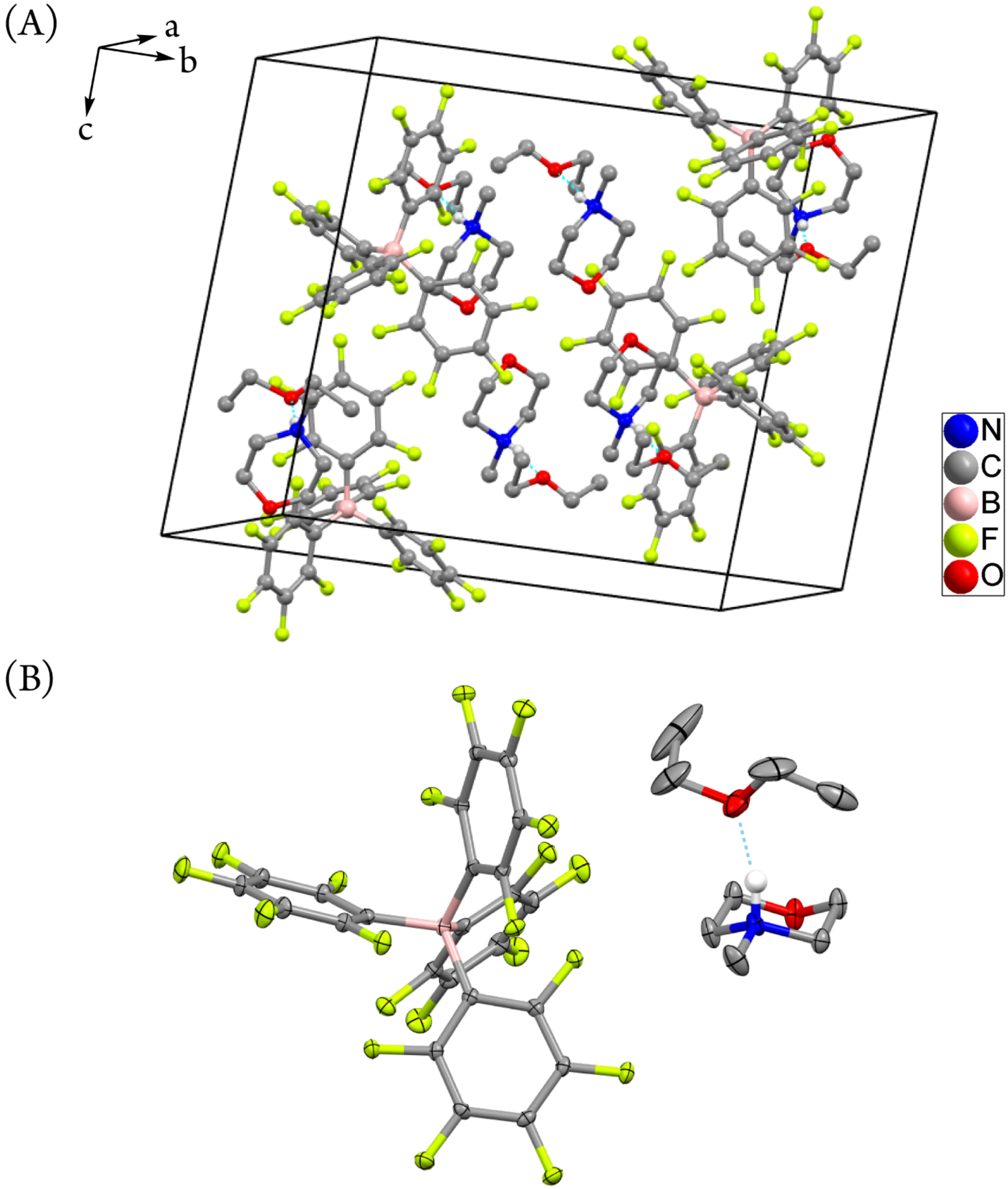

Figure S33. Diagram illustrating the (A) molecular packing and (B) displacement ellipsoid plot (50% probability level) of [4-MeMorphH][$B(C_6F_5)_4$]·Et_2O at 100(2) K. All of the hydrogens except for the acidic proton have been omitted for clarity. Dotted lines show the H-bonding interactions between the protonated nitrogen species and diethyl ether molecules.

(A)

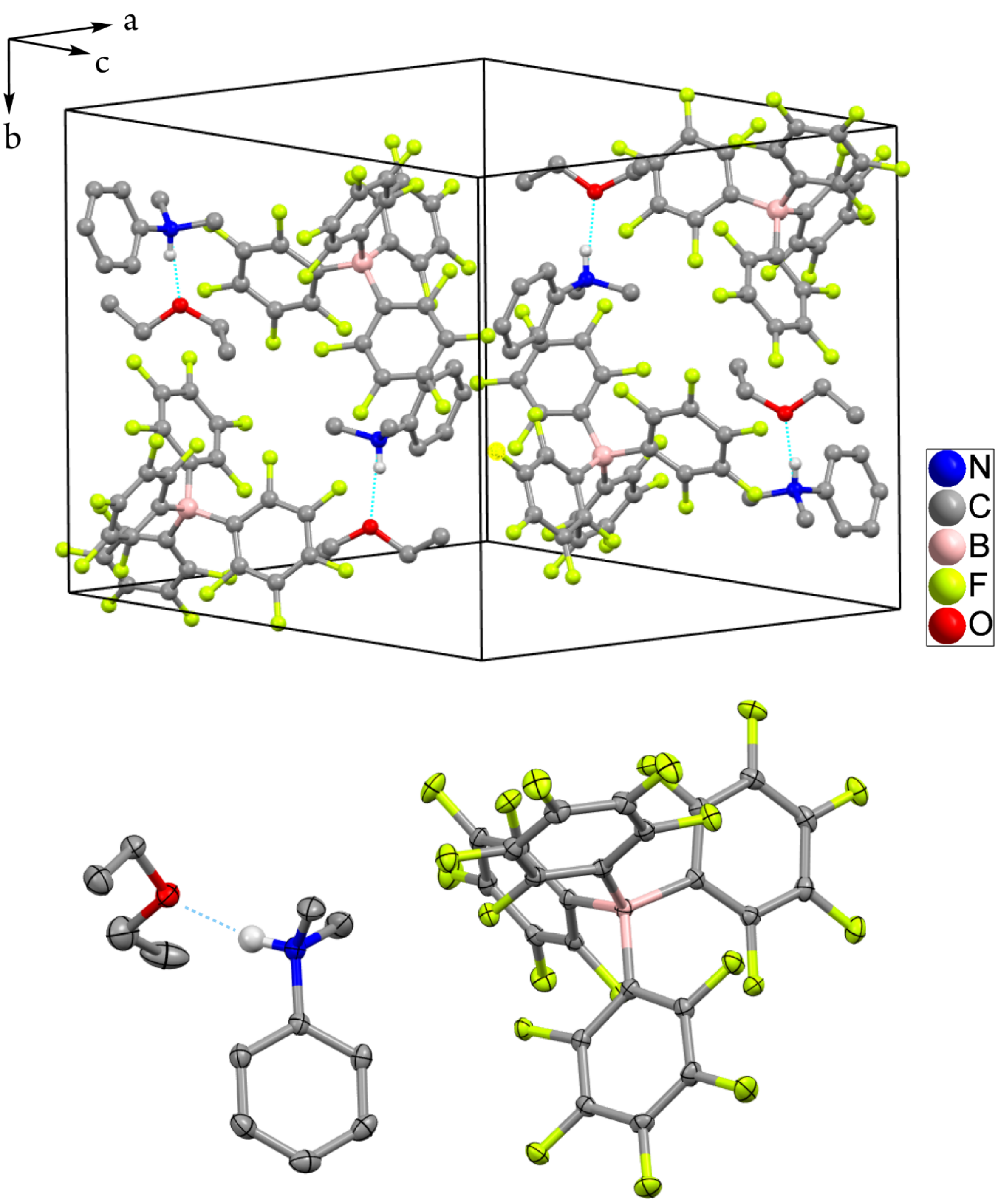

(B)

Figure S34. Diagram illustrating the (A) molecular packing and (B) displacement ellipsoid plot (50% probability level) of $[PhMe_2NH][B(C_6F_5)_4]\cdot Et_2O$ at 100(2) K. All of the hydrogens except for the acidic proton have been omitted for clarity. Dotted lines show the H-bonding interactions between the protonated nitrogen species and diethyl ether molecules.

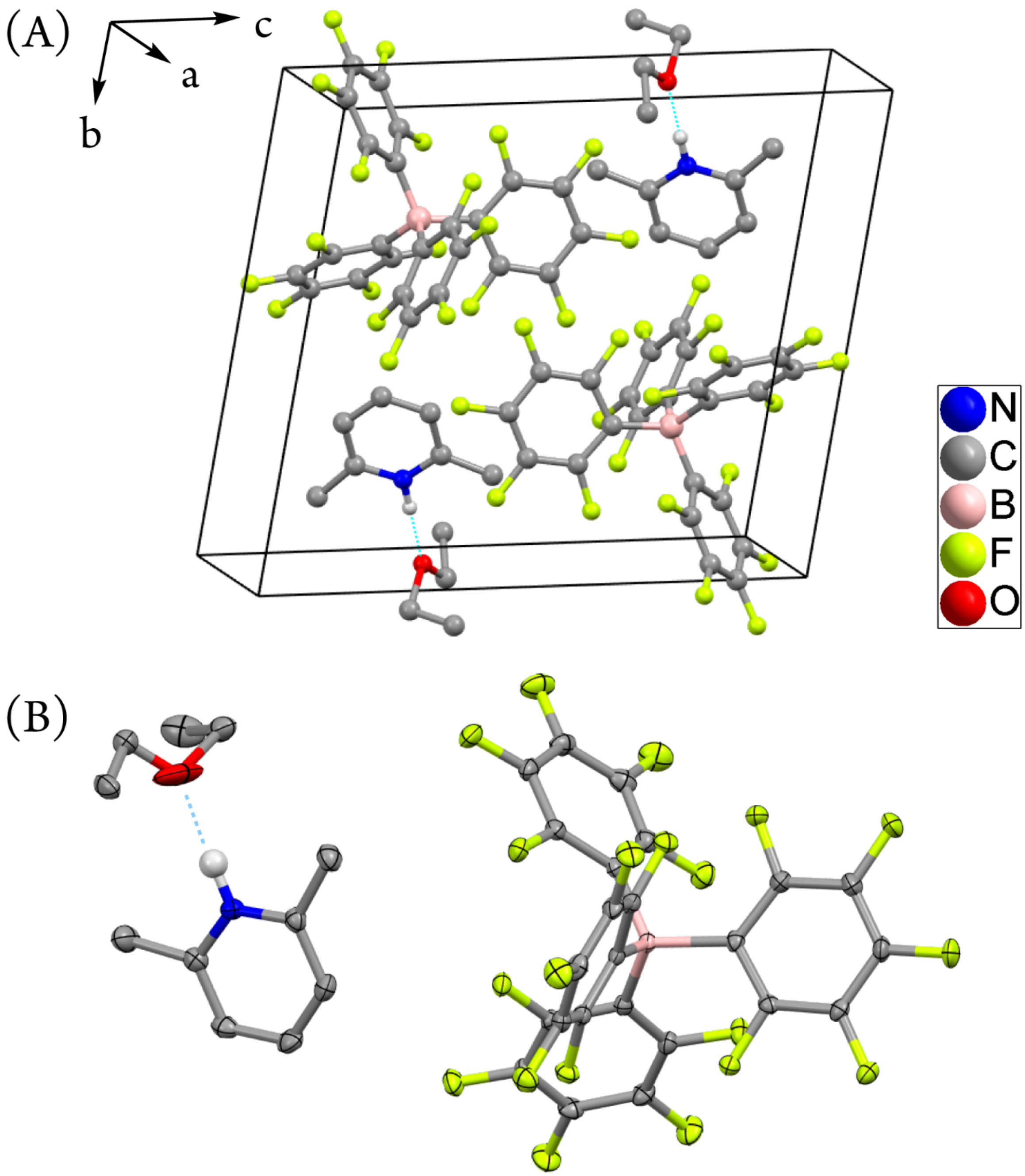

Figure S35. Diagram illustrating the (A) molecular packing and (B) displacement ellipsoid plot (50% probability level) of [2,6-Me_2PyH][B(C_6F_5)$_4$]·Et_2O at 100(2) K. All of the hydrogens except for the acidic proton have been omitted for clarity. Dotted lines show the H-bonding interactions between the protonated nitrogen species and diethyl ether molecules.

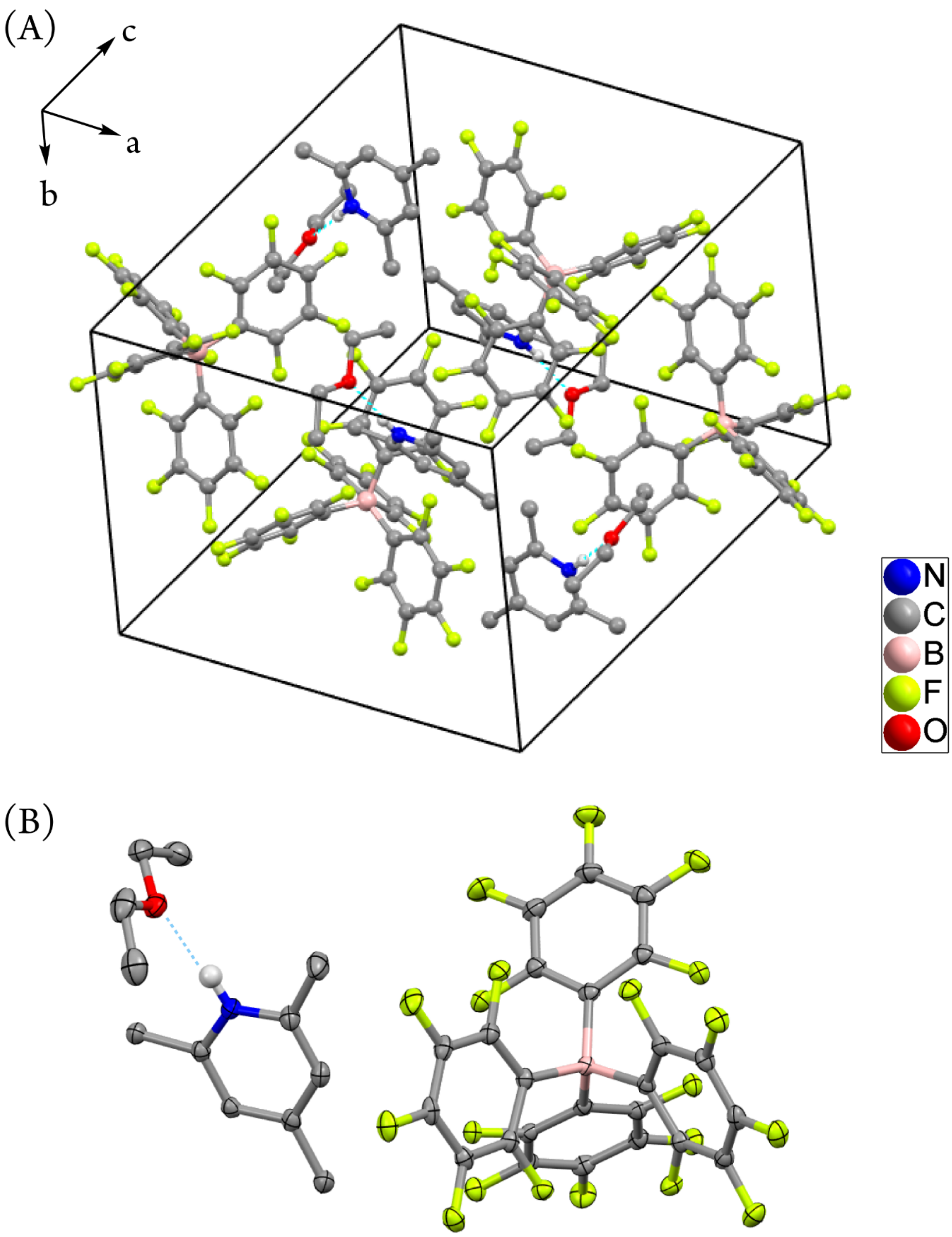

Figure S36. Diagram illustrating the (A) molecular packing and (B) displacement ellipsoid plot (50% probability level) of [2,4,6-Me_3PyH][B(C_6F_5)$_4$]·Et_2O at 100(2) K. All of the hydrogens except for the acidic proton have been omitted for clarity. Dotted lines show the H-bonding interactions between the protonated nitrogen species and diethyl ether molecules.

Table S2. Crystallographic Data and Data Collection Parameters.

	$[PhMe_2NH][B(C_6F_5)_4]\cdot Et_2O$	$[2,4,6\text{-}Me_3PyH][B(C_6F_5)_4]\cdot Et_2O$	$[2,6\text{-}Me_3PyH][B(C_6F_5)_4]\cdot Et_2O$
Formula	$C_{36}H_{22}BF_{20}NO$	$C_{36}H_{22}BF_{20}NO$	$C_{35}H_{20}BF_{20}NO$
T (K)	100(2)	100(2)	100(2)
Formula weight	875.35	875.35	861.33
Crystal system	Monoclinic	Monoclinic	Triclinic
Space group	$P\,2_1/n$	$P\,2_1/n$	*P* -1
a, Å	14.9558(2)	14.4017(2)	8.9116(4)
b, Å	15.6222(2)	16.2969(2)	13.3322(5)
c, Å	16.5644(3)	16.1025(3)	14.7542(5)
α , deg	90	90	101.560(3)
β, deg	114.105(2)	106.786(2)	96.514(3)
γ, deg	90	90	90.220(3)
V, Å3	3532.66(10)	3618.27(10)	1705.68(12)
Z	4	4	2
Radiation (λ, Å)	CuKα (1.54178)	CuKα (1.54178)	CuKα (1.54178)
*d*calcd, g•cm^{-3}	1.646	1.607	1.677
F(000)	1752	1752	860
Crystal size (mm^3)	0.502 x 0.257 x 0.168	0.404 x 0.319 x 0.147	0.279 x 0.221 x 0.062
Theta range for data collection	3.361 to 71.900°	3.631 to 71.892°	3.078 to 71.898°
μ, mm^{-1}	1.555	1.518	1.599
No of unique data	6912	7069	6617
Completeness to theta	99.7%	99.7%	99.2%
No. of restraints	0	0	0
No. of params. refined	569	541	579
GOF on F^2	1.050	1.026	1.009
$R1^{a\,[I>2\sigma(I)]}$	0.0317	0.0392	0.0393
$R1^{a}$ (all data)	0.0339	0.0425	0.0562
$wR2^{b}$ (all data)	0.0870	0.1113	0.1068
Largest diff. peak and hole	0.340 and −0.226 e.Å^{-3}	0.345 and -0.280 e.Å^{-3}	0.388 and -0.253 e.Å^{-3}

$^{a}R1 = \frac{\Sigma||F_o|-|F_c||}{\Sigma|F_o|}$; $^{b}wR2 = \sqrt{\frac{\Sigma\left[w\left(F_o{}^2-F_c{}^2\right)^2\right]}{\Sigma\left[w\left(F_o{}^2\right)^2\right]}}$

Table S3. Crystallographic Data and Data Collection Parameters.

	[4-MeMorphH][B(C_6F_5)$_4$]·Et_2O	[Me_3NH][B(C_6F_5)$_4$]·Et_2O	[Et_3NH][B(C_6F_5)$_4$]·Et_2O
Formula	$C_{33}H_{22}BF_{20}NO_2$	$C_{31}H_{20}BF_{20}NO$	$C_{34}H_{26}BF_{20}NO$
T (K)	100(2)	100(2)	100(2)
Formula weight	855.32	813.29	855.37
Crystal system	Monoclinic	Monoclinic	Orthorhombic
Space group	*P* 2_1/n	*P* 2_1/n	*P*bca
a, Å	10.6017(2)	14.7769(2)	19.1157(2)
b, Å	21.1391(4)	14.3967(2)	16.9019(2)
c, Å	16.9979(2)	16.3053(2)	21.2863(2)
α , deg	90	90	90
β, deg	106.165(2)	111.528(2)	90
γ, deg	90	90	90
V, Å3	3658.80(11)	3226.78(8)	6877.43(13)
Z	4	4	8
Radiation (*λ*, Å)	CuKα (1.54178)	CuKα (1.54178)	CuKα (1.54178)
*d*calcd, g•cm^{-3}	1.553	1.674	1.652
F(000)	1712	1624	3440
Crystal size (mm^3)	0.371 x 0.244 x 0.169	0.627 x 0.513 x 0.244	0.500 x 0.343 x 0.151
Theta range for data collection	3.420 to 71.900°	3.457 to 71.895°	4.062 to 71.898°
μ, mm^{-1}	1.505	1.646	1.576
No of unique data	7153	6308	6735
Completeness to theta	100.0%	99.6%	99.8%
No. of restraints	0	0	0
No. of params. refined	574	496	523
GOF on F^2	1.020	1.047	1.046
$R1^{a}$ [I> 2σ(I)]	0.0344	0.0390	0.0349
$R1^{a}$ (all data)	0.0445	0.0412	0.0376
$wR2^{b}$ (all data)	0.0882	0.1072	0.0959
Largest diff. peak and hole	0.348 and -0.306 e.Å^{-3}	0.395 and -0.294 e.Å^{-3}	0.688 and -0.367 e.Å^{-3}

$^{a}R1= \frac{\Sigma||F_o|-|F_c||}{\Sigma|F_o|}$; $^{b}wR2= \sqrt{\frac{\Sigma[w(F_o{}^2-F_c{}^2)^2]}{\Sigma[w(F_o{}^2)^2]}}$

Table S4. Selected Bond Lengths (Å) and Angles (deg) for Nitrogen Bases and Their Protonated Counterparts.

Compound	C–N (Å)		∠C–N–C (°)		Ref. for N-base
	N-base	N-acid	N-base	N-acid	
Et_3N/H^+	1.471<br>1.475<br>1.481	1.508<br>1.507<br>1.507	113.1<br>111.3<br>111.0	113.2<br>113.1<br>110.6	d
Me_3N/H^+	1.448<br>1.448<br>1.448	1.494<br>1.488<br>1.487	110.7<br>110.7<br>110.6	111.3<br>111.0<br>110.4	e
4-MeMorph/H^+	1.471<br>1.467<br>1.460	1.502<br>1.491<br>1.489	110.9<br>109.8<br>107.9	112.2<br>111.8<br>109.3	f
2,4,6-Me_3Py/H^+	1.348<br>1.343	1.353<br>1.352	118.1	123.6	g
2,6-Me_2Py/H^+	1.352<br>1.346	1.351<br>1.343	120.0	124.6	h
$PhMe_2N/H^+$	1.441<br>1.426<br>1.390	1.500<br>1.497<br>1.479	120.1<br>119.8<br>118.7	114.0<br>110.8<br>110.6	i

IR Spectroscopy

Table S5. Comparison of N–H Stretching Frequencies for Protonated Nitrogen Bases Described in This Study.

Compound	$\upsilon_{(N-H)}$ (cm^{-1})	Compound	$\upsilon_{(N-H)}$ (cm^{-1})
$[Et_3NH]^+$	3241	$[2,4,6\text{-}Me_3PyH]^+$	3367
$[Me_3NH]^+$	3356	$[2,6\text{-}Me_2PyH]^+$	3356
$[BnNH_3]^+$	3306	$[2\text{-}MePyH]^+$	3358
$[4\text{-}MeMorphH]^+$	3238	$[PhMe_2NH]^+$	3250

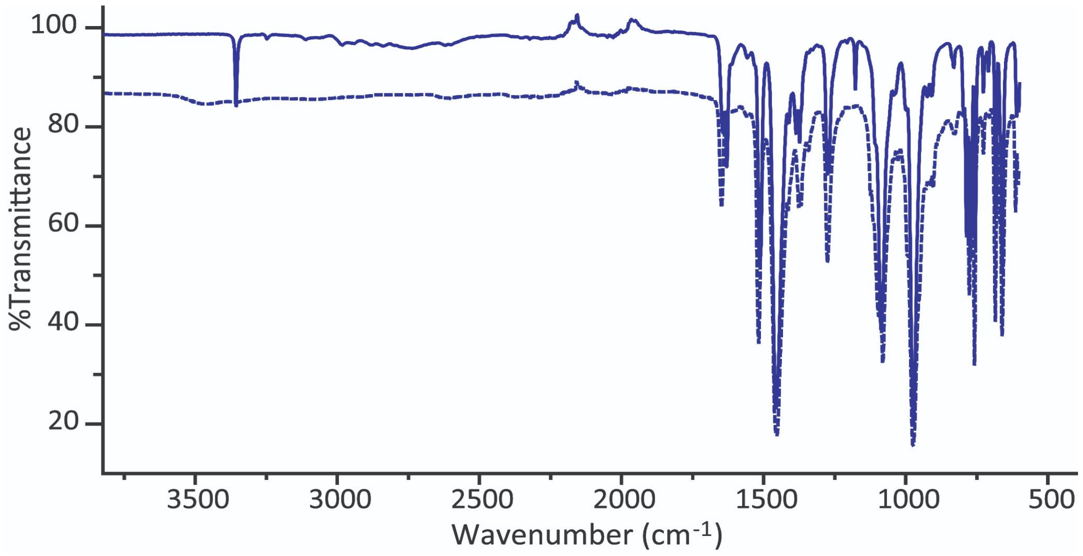

Figure S37. FT-IR spectra of $K[B(C_6F_5)_4]$ (dotted line) and $[Me_3NH][B(C_6F_5)_4]$ (solid line).

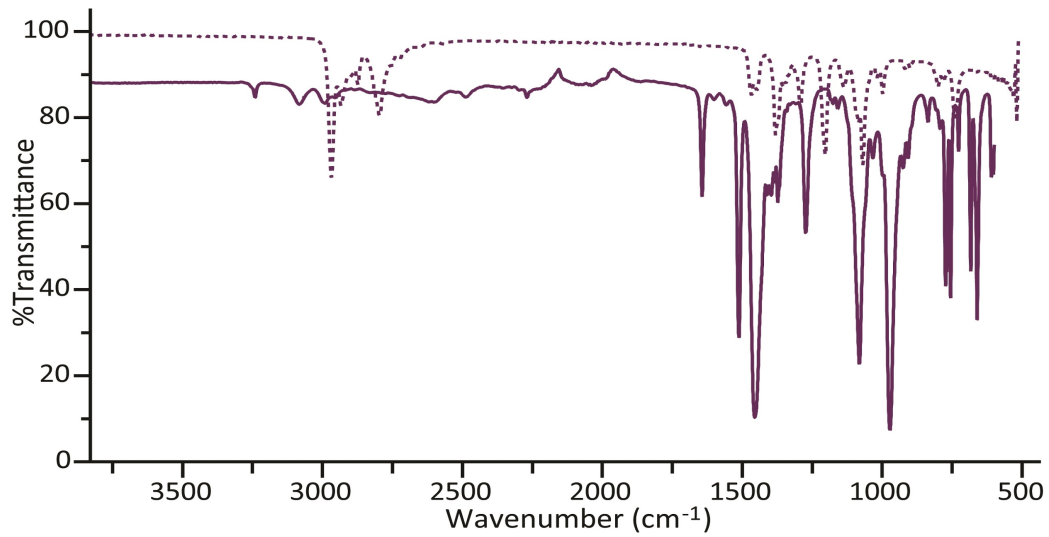

Figure S38. FT-IR spectral comparison of Et_3N (dotted line) and $[Et_3NH][B(C_6F_5)_4]$ (solid line).

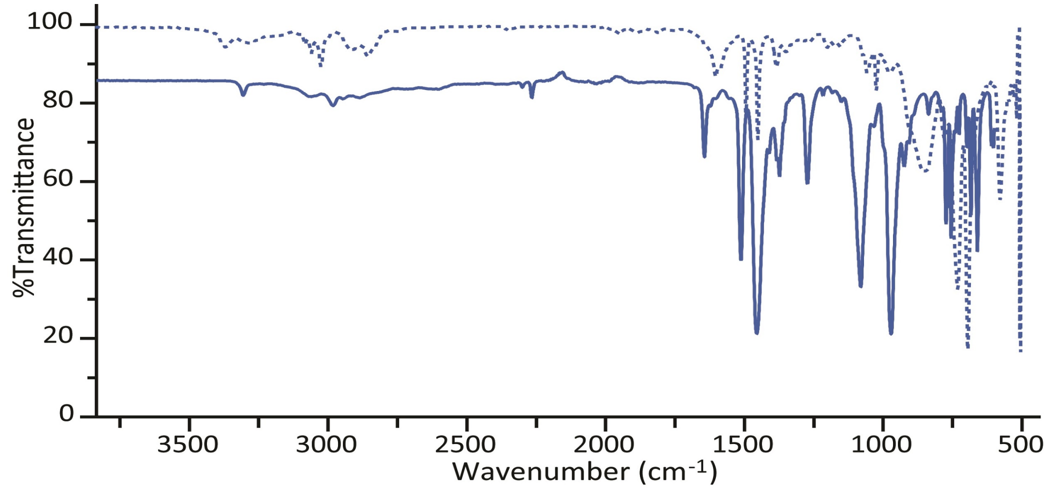

Figure S39. FT-IR spectral comparison of $BnNH_2$ (dotted line) and $[BnNH_3][B(C_6F_5)_4]$ (solid line).

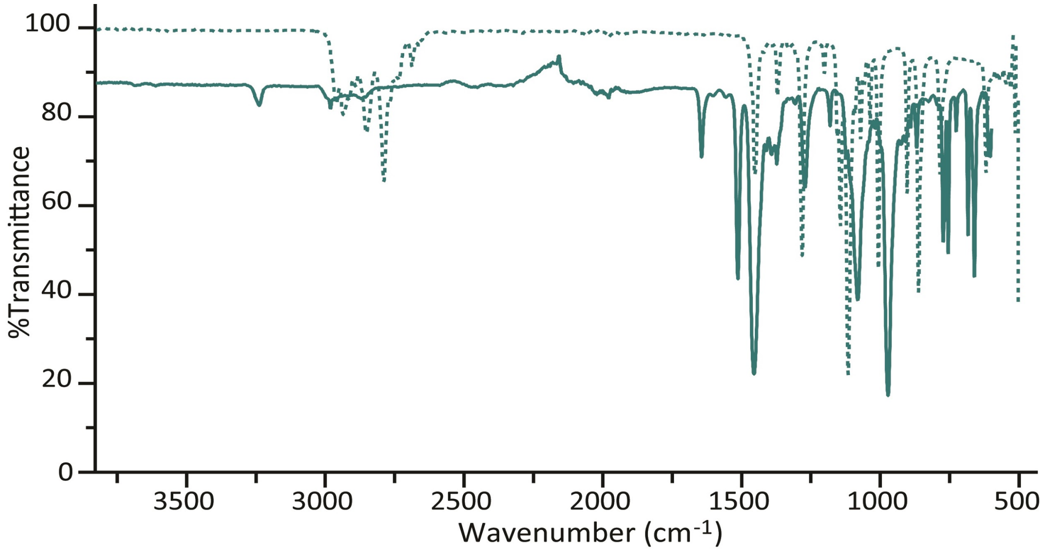

Figure S40. FT-IR spectral comparison of 4-MeMorph (dotted line) and $[4\text{-MeMorphH}][B(C_6F_5)_4]$ (solid line).

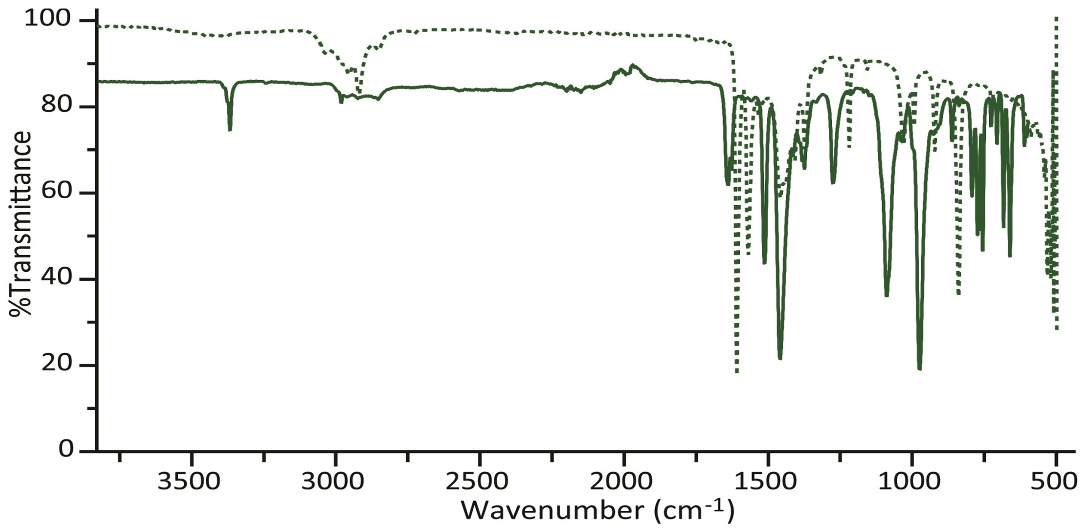

Figure S41. FT-IR spectral comparison of 2,4,6-Me_3Py (dotted line) and [2,4,6-Me_3PyH][$B(C_6F_5)_4$] (solid line).

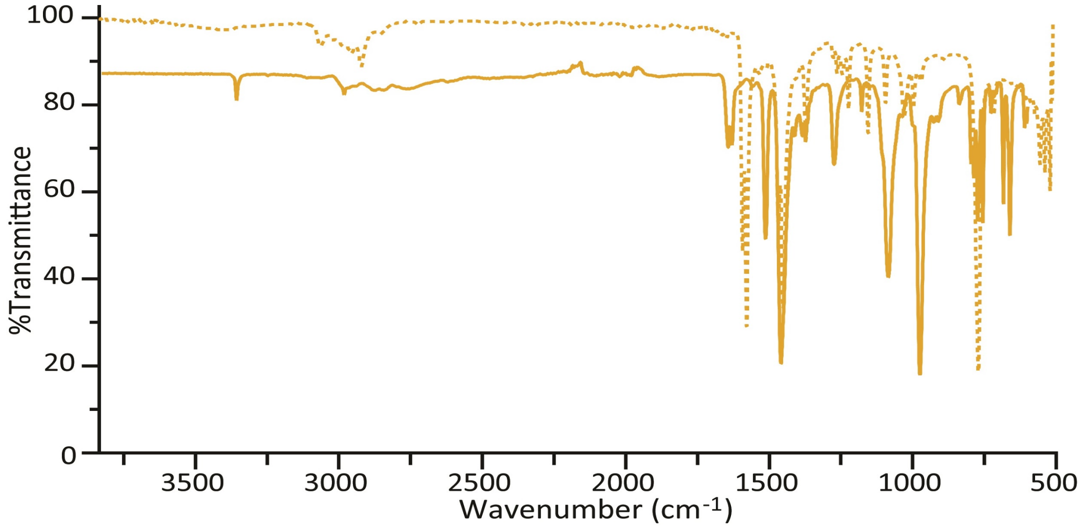

Figure S42. FT-IR spectral comparison of 2,6-Me_2Py (dotted line) and [2,6-Me_2PyH][$B(C_6F_5)_4$] (solid line).

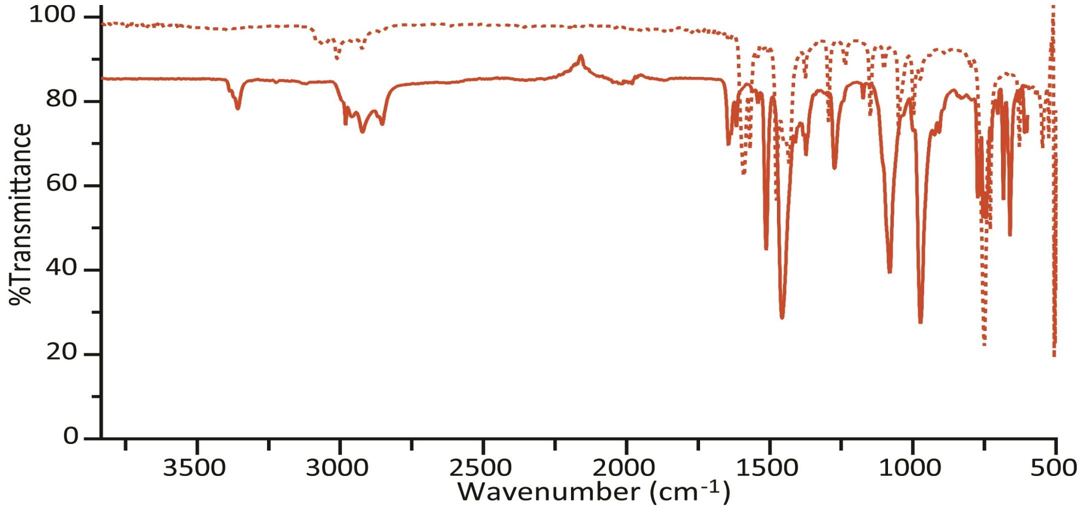

Figure S43. FT-IR spectral comparison of 2-MePy (dotted line) and [2-MePyH][$B(C_6F_5)_4$] (solid line).

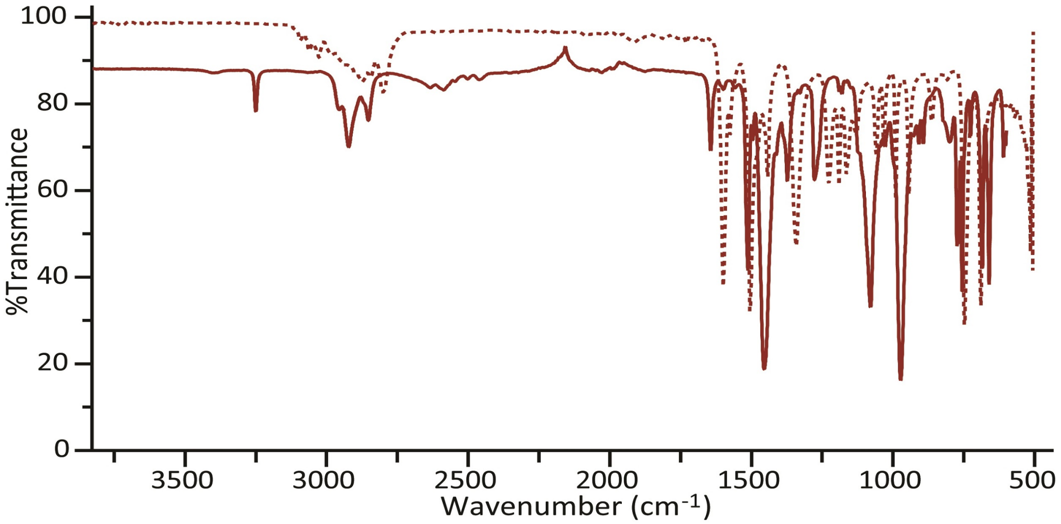

Figure S44. FT-IR spectral comparison of $PhMe_2N$ (dotted line) and $[PhMe_2NH][B(C_6F_5)_4]$ (solid line).

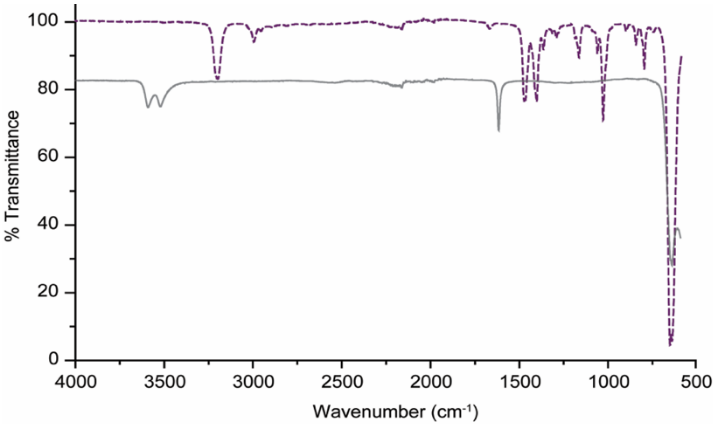

Figure S45. FT-IR spectral comparison of $Ag[SbF_6]$ (solid line) and $[Et_3NH][SbF_6]$ (dotted line).

a. Meetsma, A. CSD Private Communication (CCDC 290023), **2005**.

b. Osi, A. Chardon, A. Tumanov, N. Wouters, J. Berionni, G. CSD Private Communication (CCDC 2120883), **2021**.

c. Sheldrick, G. M. *SHELXL-2018: Program for Crystal Structure Refinement*, University of Göttingen, Göttingen, Germany, **2018**.

d. Klien, H.; Seichter, W.; Weber, E., Crystal Structure of 2,2″-Bis-(2,7-di-chloro-9-hy-dr-oxy- 9H-fluoren-9-yl)-1,1′:4′,1″-terphenyl Tri-ethyl-amine Tris-olvate. *Acta Cryst. E* **2015,** *71*, 1439-1443.

e. Boese, R.; Bläser, D.; Y. Antipin, M.; Boese, R.; Y. Antipin, M.; Chaplinski, V.; de Meijere, A., Non-planar Structures of Et_3N and $Pr^{i}_{3}N$: A Contradiction between the X-ray, and NMR and Electron Diffraction Data for $Pr^{i}_{3}N$. *Chem. Commun.* **1998**, 781-782.

f. Oswald, I. D. H.; Motherwell, W. D. S.; Parsonsa, S., Formation of Quinol Co-crystals with Hydrogen-bond Acceptors. *Acta Crystallogr. Sect. B: Struct. Sci.* **2005,** *61*, 46-57.

g. Jiang, X.; O'Brien, Z. J.; Yang, S.; Lai, L. H.; Buenaflor, J.; Tan, C.; Khan, S.; Houk, K. N.; Garcia-Garibay, M. A., Crystal Fluidity Reflected by Fast Rotational Motion at the Core, Branches, and Peripheral Aromatic Groups of a Dendrimeric Molecular Rotor. *J. Am. Chem. Soc.* **2016,** *138* (13), 4650-4656.

h. Schmidtmann, M.; Wilson, C. C., Hydrogen Transfer in Pentachlorophenol – Dimethylpyridine Complexes. *CrystEngComm* **2008,** *10* (2), 177-183.

i. Schwenger, A.; Frey, W.; Richert, C., Reagents with a Crystalline Coat. *Angew. Chem. Int. Ed.* **2016,** *55* (44), 13706-13709.
